# World Health Organization Estimates of the Global and Regional Disease Burden of 11 Foodborne Parasitic Diseases, 2010: A Data Synthesis

**DOI:** 10.1371/journal.pmed.1001920

**Published:** 2015-12-03

**Authors:** Paul R. Torgerson, Brecht Devleesschauwer, Nicolas Praet, Niko Speybroeck, Arve Lee Willingham, Fumiko Kasuga, Mohammad B. Rokni, Xiao-Nong Zhou, Eric M. Fèvre, Banchob Sripa, Neyla Gargouri, Thomas Fürst, Christine M. Budke, Hélène Carabin, Martyn D. Kirk, Frederick J. Angulo, Arie Havelaar, Nilanthi de Silva

**Affiliations:** 1 University of Zürich, Zürich, Switzerland; 2 Ghent University, Ghent, Belgium; 3 Université catholique de Louvain, Brussels, Belgium; 4 Institute of Tropical Medicine, Antwerp, Belgium; 5 Ross University School of Veterinary Medicine, St. Kitts, West Indies; 6 National Institute of Health Sciences, Tokyo, Japan; 7 Tehran University of Medical Sciences, Tehran, Iran; 8 Chinese Center for Disease Control and Prevention, Shanghai, People’s Republic of China; 9 University of Liverpool, Liverpool, United Kingdom; 10 International Livestock Research Institute, Nairobi, Kenya; 11 Khon Kaen University, Khon Kaen, Thailand; 12 Hikma Pharmaceuticals, Amman, Jordan; 13 Imperial College, London, United Kingdom; 14 Texas A&M University, College Station, Texas, United States of America; 15 University of Oklahoma Health Sciences Center, Oklahoma City, Oklahoma, United States of America; 16 The Australian National University, Canberra, Australia; 17 Centers for Disease Control and Prevention, Atlanta, Georgia, United States of America; 18 National Institute for Public Health and the Environment, Bilthoven, The Netherlands; 19 Utrecht University, Utrecht, The Netherlands; 20 University of Florida, Gainesville, Gainesville, Florida, United States of America; 21 University of Kelaniya, Ragama, Sri Lanka; Mahidol-Oxford Tropical Medicine Research Unit, THAILAND

## Abstract

**Background:**

Foodborne diseases are globally important, resulting in considerable morbidity and mortality. Parasitic diseases often result in high burdens of disease in low and middle income countries and are frequently transmitted to humans via contaminated food. This study presents the first estimates of the global and regional human disease burden of 10 helminth diseases and toxoplasmosis that may be attributed to contaminated food.

**Methods and Findings:**

Data were abstracted from 16 systematic reviews or similar studies published between 2010 and 2015; from 5 disease data bases accessed in 2015; and from 79 reports, 73 of which have been published since 2000, 4 published between 1995 and 2000 and 2 published in 1986 and 1981. These included reports from national surveillance systems, journal articles, and national estimates of foodborne diseases. These data were used to estimate the number of infections, sequelae, deaths, and Disability Adjusted Life Years (DALYs), by age and region for 2010. These parasitic diseases, resulted in 48.4 million cases (95% Uncertainty intervals [UI] of 43.4–79.0 million) and 59,724 (95% UI 48,017–83,616) deaths annually resulting in 8.78 million (95% UI 7.62–12.51 million) DALYs. We estimated that 48% (95% UI 38%-56%) of cases of these parasitic diseases were foodborne, resulting in 76% (95% UI 65%-81%) of the DALYs attributable to these diseases. Overall, foodborne parasitic disease, excluding enteric protozoa, caused an estimated 23.2 million (95% UI 18.2–38.1 million) cases and 45,927 (95% UI 34,763–59,933) deaths annually resulting in an estimated 6.64 million (95% UI 5.61–8.41 million) DALYs. Foodborne *Ascaris* infection (12.3 million cases, 95% UI 8.29–22.0 million) and foodborne toxoplasmosis (10.3 million cases, 95% UI 7.40–14.9 million) were the most common foodborne parasitic diseases. Human cysticercosis with 2.78 million DALYs (95% UI 2.14–3.61 million), foodborne trematodosis with 2.02 million DALYs (95% UI 1.65–2.48 million) and foodborne toxoplasmosis with 825,000 DALYs (95% UI 561,000–1.26 million) resulted in the highest burdens in terms of DALYs, mainly due to years lived with disability. Foodborne enteric protozoa, reported elsewhere, resulted in an additional 67.2 million illnesses or 492,000 DALYs. Major limitations of our study include often substantial data gaps that had to be filled by imputation and suffer from the uncertainties that surround such models. Due to resource limitations it was also not possible to consider all potentially foodborne parasites (for example *Trypanosoma cruzi*).

**Conclusions:**

Parasites are frequently transmitted to humans through contaminated food. These estimates represent an important step forward in understanding the impact of foodborne diseases globally and regionally. The disease burden due to most foodborne parasites is highly focal and results in significant morbidity and mortality among vulnerable populations.

## Abstract 2

In this data synthesis, Paul Robert Torgerson and colleagues estimate the global and regional disease burden of 11 foodborne parasitic diseases.

## Editors' Summary

**Background:**

Foodborne diseases cause a large burden of illness (morbidity) and death (mortality), worldwide. More than 200 diseases can be transmitted to people through the ingestion of food contaminated by microorganisms (viruses, bacteria, and parasites) or with chemicals. Food can become contaminated on the farms where crops and animals are raised, in food processing plants, and during food storage and preparation at home and in restaurants. Food contamination can be caused by pollution of water and soil by human and animal feces and by poor hygiene practices such as not washing one’s hands after using the toilet or before handling food. Many foodborne diseases present with gastrointestinal symptoms (stomach cramps, diarrhea and vomiting) but some also affect other parts of the body and some have serious sequelae (abnormal bodily conditions or diseases arising from a pre-existing disease). For example, the parasitic tapeworm *Taenia solium* (which is spread by eating undercooked pork) can cause cysticercosis, an infection of tissues by larval cysts that can result in seizures, stroke and death.

**Why Was This Study Done?:**

National and international efforts to improve food safety need accurate information on foodborne infections so, in 2007, the World Health Organization (WHO) established the Foodborne Disease Burden Epidemiology Reference Group (FERG) to provide estimates of the global and regional burden of disease attributable to foodborne illnesses. Here, researchers involved in one of FERG’s constituent task forces—the Parasitic Diseases Task Force—combine information from many different sources (a data synthesis) to provide estimates of the regional and global disease burden of ten helminth diseases and toxoplasmosis attributable to contaminated food. Examples of helminths (parasitic worms) include roundworms (*Ascaris lumbricoides*; heavy roundworm infections [ascariosis] can cause signs of malnutrition or even intestinal blockages), tapeworms and flukes (liver and lung flukes cause a condition known as trematodosis; frequently transmitted in undercooked fish crustacea or aquatic vegetables). Toxoplasmosis is caused by a parasite found in undercooked meat and in cat feces. If a woman becomes infected during pregnancy, she can pass the parasite onto her unborn child (congenital toxoplasmosis), thereby causing eye problems and sometimes developmental problems and seizures later in life.

**What Did the Researchers Do and Find?:**

The researchers combined national estimates of foodborne diseases, and data from systematic reviews (studies that identify all the research on a topic using predefined criteria), national surveillance programs, and other sources to estimate the number of illnesses, sequelae, and deaths for ten helminth diseases and toxoplasmosis. They also estimated the number of disability adjusted life years (DALYs) globally and regionally for each disease. A DALY is the disease-related loss of one year of full health because of premature death or disability; DALYs provide a measure of the burden of a disease. Together, these diseases caused 48.4 million cases of illness, 59,724 deaths, and 8.78 million DALYs in 2010. The researchers estimated that 48% of these cases of parasitic diseases, resulting in 6.64 million DALYs, were transmitted by contaminated food. The commonest foodborne parasitic diseases were *Ascaris* infection and toxoplasmosis (12.3 million and 10.3 million cases, respectively). Foodborne cysticercosis, trematodosis and toxoplasmosis resulted in the highest disease burdens, and the largest burden of foodborne parasitic disease occurred in the Western Pacific and African regions.

**What Do These Findings Mean?:**

The lack of reliable data on the diseases considered in this study for many regions of the world and the use of expert panels to estimate the proportion of each disease that is foodborne may limit the accuracy of these findings. Moreover, this study does not estimate the global burden of every potentially important foodborne parasitic disease. However, these findings, together with those on three foodborne enteric protozoa (single-celled parasites that infect the intestines) included in a related paper, indicate that parasites are frequently transmitted to people through contaminated food and that, although some parasites result in a low burden of disease, foodborne parasites result in significant illness and death that is often borne by relatively small populations in limited geographical areas. This information, together with other estimates on foodborne disease obtained by FERG, should facilitate the development and implementation of effective national and global food safety policies.

**Additional Information:**

This list of resources contains links that can be accessed when viewing the PDF on a device or via the online version of the article at http://dx.doi.org/10.1371/journal.pmed.1001920.

- A related *PLOS Medicine* Research Article by Kirk et al that includes data on foodborne enteric parasites is available
- The World Health Organization provides information about soil-transmitted helminths, foodborne diseases, food safety, and the estimation of the global burden of foodborne diseases (available in several languages); it also provides fact sheets on cysticercosis, trematodosis, and ascariasis
- The US Centers for Disease Control and Prevention (CDC) provides information about foodborne disease outbreaks in the US and elsewhere and information about food safety in the US; it also provides general information about soil-transmitted helminths; more detailed information about individual diseases caused by helminths and about toxoplasmosis can be found by visiting the CDC’s alphabetical index of parasites
- The UK National Health Service Choices website provides information about roundworm infections and tapeworm infections, and about food safety
- PARA-SITE, an electronic resource devoted to parasitology that is provided by the Australian Society of Parasitology, provides more information about the biology of helminth parasites

## Introduction

Foodborne diseases are an important public health problem worldwide [1,2]. Reliable epidemiological estimates on the burden of foodborne diseases are important to assess the potential impact of food safety measures and advise policy-makers on the cost-effective use of often scarce resources. To date, however, no precise and consistent global information exists on most agents or pathogens transmitted by contaminated food. In particular, many pathogens that may be transmitted by food are often neglected and affect vulnerable and marginalized populations where the burden may be high [3].

Knowledge of the agent-specific burden of foodborne diseases can assist policy makers to improve food safety. In 2007 the World Health Organization (WHO) established the Foodborne Disease Burden Epidemiology Reference Group (FERG) to estimate global and regional burdens of foodborne disease [4]. The FERG established three thematic task forces to estimate the burden of foodborne disease due to (1) chemicals, (2) enteric viruses and bacteria, and (3) parasites. In this study the Parasitic Diseases Task Force (PDTF) reports estimates of the burden of 11 parasitic diseases and the estimated proportion of this burden that is transmitted by contaminated food. The burden of a further three parasitic diseases: cryptosporidiosis, entamoebosis and giardiosis, were also estimated. These are detailed in a report by Kirk et al on enteric pathogens [5], however summary information on these 3 pathogens is also reported here to complete the picture of the burden of foodborne parasitic diseases.

## Methods

Following a public call for advisers in the scientific press and a transparent selection process, the WHO Director-General appointed the FERG members from a large pool of applicants.

FERG members include scientists with outstanding international reputations in food sciences, epidemiology, veterinary sciences, medical sciences, microbiology, chemical and other risk assessment, food policy and regulation, statistics and geographic information systems, among others. The PDTF consisted of FERG members who, within this group, had particular expertise in parasitology. There were in total 10 scientists who were members of the PDTF at some point during the study representing institutes from China, Denmark, Iran, Japan, Jordan, Kenya, Sri Lanka, Switzerland, Thailand and the UK.

At the first formal meeting of FERG, the PDTF initially reviewed all parasitic diseases that could be potentially transmitted by food with14 parasitic diseases selected as high priority (Table 1). The selection criteria of these 14 diseases was based on: proportion of foodborne transmission; severity of illness and/or sequelae; frequency of illness and/or sequelae causes; global relevance; particular regional relevance; propensity to cause outbreaks, and availability of existing evidence to derive burden estimates [6]. Three intestinal protozoa *Cryptosporidium*, *Entamoeba* and *Giardia* spp. were considered priority as they were likely to result in a high disease burden and the frequency of citations for these parasites had been markedly increasing between 1990 and 2008 [7]. *Cyclospora* was also initially considered but a decision was made to target resources on the other intestinal protozoa as citation frequency had remained constant over the same period. For methodological reasons, the burden of the three priority intestinal protozoa that cause diarrheal disease was estimated by the Enteric Disease Task Force and are reported in more detail by Kirk et al [5]. *Toxoplasma gondii* was also considered to be of high priority because of the potential serious sequelae. Foodborne trematodes of high priority were *Fasciola* spp., *Clonorchis* spp., *Opisthorchis* spp., *Paragonimus* spp. and intestinal trematodes such as *Fasciolopsis buski*, *Heterophyes* spp. and *Metagonimus* spp. Three cestode species were considered important: *Echinococcus granulosus*, *E*. *multilocularis* and *Taenia solium*. The cestode *Taenia saginata* was considered likely to have a very low burden to human health because of the lack of serious sequelae resulting from intestinal taeniosis and hence was excluded from the priority list. Foodborne Chagas disease was also considered for possible inclusion at the second FERG meeting [7], but resources were not available to commission work on the foodborne transmission of this disease. Finally the nematode species believed to have high impact were Anisakidae, *Ascaris lumbricoides* and *Trichinella* spp. Disease caused by the Anisakidae was later considered to be an uncommon foodborne disease and was subsequently removed from the priority list. In this paper, the PDTF reports in detail the disease burden of the remaining 11 diseases with summary information on the three enteric protozoa. For consistency, the standardised nomenclature on parasitic diseases [8] is used throughout the manuscript. For a glossary of terms used in this manuscript, including the regions, see S1 Text.

**Table 1:** Median number of total and foodborne illnesses, deaths, and Disability Adjusted Life Years (DALYs), with 95% uncertainty intervals, 2010.

PATHOGEN	ILLNESSES (95% UI)	DEATHS (95% UI)	DALYs (95% UI)	PROPORTION FOODBORNE—ILLNESSES (95% UI)	PROPORTION FOODBORNE—DALYs (95% UI)	FOODBORNE ILLNESSES (95% UI)	FOODBORNE DEATHS (95% UI)	FOODBORNE DALYS (95% UI)
**Enteric protozoa** ^**#**^	356,688,438 (251,929,267–517,872,930)	33,925 (22,933–53,614)	2,940,604 (1,978,442–4,686,855)	0.19 (0.12–0.28)	0.17 (0.09–0.29)	67,182,645 (35,794,977–120,556,797)	5,558 (2,593–11,958)	492,354 (239,400–1,034,790)
*Cryptosporidium* spp.^**#**^	64,003,709 (43,049,455–104,679,951)	27,553 (18,532–44,654)	2,159,331 (1,392,438–3,686,925)	0.13 (0.07–0.24)	0.14 (0.06–0.28)	8,584,805 (3,897,252–18,531,196)	3,759 (1,520–9,115)	296,156 (119,456–724,660)
*Entamoeba histolytica* ^**#**^	103,943,952 (47,018,659–210,632,459)	5,450 (2,194–17,127)	515,904 (222,446–1,552,466)	0.28 (0.14–0.44)	0.28 (0.13–0.47)	28,023,571 (10,261,254–68,567,590)	1,470 (453–5,554)	138,863 (47,339–503,775)
*Giardia* spp.^**#**^	183,842,615 (130,018,020–262,838,002)	0 (0–0)	171,100 (115,777–257,315)	0.15 (0.08–0.27)	0.15 (0.07–0.27)	28,236,123 (12,945,655–56,996,454)	0 (0–0)	26,270 (11,462–53,577)
**Invasive infectious disease**	20,817,916 (16,337,908–29,091,701)	1,409 (701–2,620)	1,684,414 (1,236,005–2,452,060)	0.49 (0.40–0.59)	0.49 (0.40–0.59)	10,280,089 (7,403,516–14,904,324)	684 (333–1,300)	829,071 (561,297–1,264,567)
*Toxoplasma gondii*, congenital	98,900 (67,858–188,748)	1,409 (701–2,620)	526,515 (359,756–835,537)	0.49 (0.40–0.58)	0.49 (0.40–0.58)	48,823 (31,893–93,213)	684 (333–1,300)	259,618 (168,510–422,935)
*Toxoplasma gondii*, acquired	20,710,906 (16,235,987–28,955,435)	0 (0–0)	1,153,779 (772,676–1,733,114)	0.49 (0.40–0.59)	0.49 (0.39–0.59)	10,228,111 (7,351,013–14,844,411)	0 (0–0)	565,816 (354,029–891,032)
**Cestodes**	596,838 (482,828–2,169,206)	48,269 (36,956–70,978)	3,721,581 (2,942,173–5,416,945)	0.72 (0.35–0.79)	0.85 (0.65–0.93)	430,864 (334,389–774,703)	36,500 (25,652–50,063)	3,158,826 (2,411,585–4,122,032)
*Echinococcus granulosus*	188,079 (156,848–1,770,405)	2,225 (749–19,627)	183,573 (88,082–1,590,846)	0.21 (0.15–0.30)	0.21 (0.15–0.29)	43,076 (25,881–371,177)	482 (150–3,974)	39,950 (16,996–322,953)
*Echinococcus multilocularis*	18,451 (11,384–29,619)	17,118 (10,184–27,346)	687,823 (409,190–1,106,320)	0.47 (0.04–0.75)	0.48 (0.01–0.76)	8,375 (656–17,005)	7,771 (243–15,896)	312,461 (9,083–640,716)
*Taenia solium*	370,710 (282,937–478,123)	28,114 (21,059–36,915)	2,788,426 (2,137,613–3,606,582)	1.00	1.00	370,710 (282,937–478,123)	28,114 (21,059–36,915)	2,788,426 (2,137,613–3,606,582)
**Nematodes**	26,845,649 (25,375,461–47,940,713)	2,227(865–5,946)	1,318,104 (1,183,025–2,701,170)	0.45 (0.31–0.59)	0.46 (0.31–0.59)	12,285,286 (8,292,732–22,984,630)	1,012 (388–2,783)	605,738 (411,113–1,301,619)
*Ascaris lumbricoides*	26,840,692 (25,371,434–47,937,154)	2,224 (862–5,942)	1,317,535 (1,182,187–2,700,572)	0.45 (0.31–0.59)	0.46 (0.31–0.59)	12,280,767 (8,287,414–22,980,491)	1,008 (384–2,781)	605,278 (410,668–1,301,114)
*Trichinella* spp.	4,472 (2,977–5,997)	4 (2–5)	550 (285–934)	1.00	1.00	4,472 (2,977–5,997)	4 (2–5)	550 (285–934)
**Trematodes**	218,569 (167,886–281,872)	7,533 (6,383–8,845)	2,024,592 (1,652,243–2,483,514)	1.00	1.00	218,569 (167,886–281,872)	7,533 (6,383–8,845)	2,024,592 (1,652,243–2,483,514)
*Clonorchis sinensis*	31,620 (21,515–45,059)	5,770 (4,728–6,988)	522,863 (431,520–635,232)	1.00	1.00	31,620 (21,515–45,059)	5,770 (4,728–6,988)	522,863 (431,520–635,232)
*Fasciola* spp.	10,635 (6,888–24,100)	0 (0–0)	90,041 (58,050–209,097)	1.00	1.00	10,635 (6,888–24,100)	0 (0–0)	90,041 (58,050–209,097)
Intestinal flukes*	18,924 (14,498–24,200)	0 (0–0)	155,165 (118,920–198,147)	1.00	1.00	18,924 (14,498–24,200)	0 (0–0)	155,165 (118,920–198,147)
*Opisthorchis* spp.	16,315 (11,273–22,860)	1,498 (1,230–1,813)	188,346 (151,906–235,431)	1.00	1.00	16,315 (11,273–22,860)	1,498 (1,230–1,813)	188,346 (151,906–235,431)
*Paragonimus* spp.	139,238 (95,610–195,078)	250 (160–371)	1,048,937 (743,700–1,438,588)	1.00	1.00	139,238 (95,610–195,078)	250 (160–371)	1,048,937 (743,700–1,438,588)
**TOTAL (excluding enteric protozoa)**	48,405,537 (43,376,746–79,049,913)	59,724 (48,017–83,616)	8,777,198 (7,620,016–12,511,566)	0.48 (0.38–0.56)	0.76 (0.65–0.81)	23,220,595 (18,215,499–38,081,817)	45,927 (34,763–59,933)	6,639,989 (5,611,955–8,414,684)
**TOTAL**	407,149,528 (301,670,420–585,323,226)	94,620 (77,140–125,670)	11,794,391 (10,164,903–15,675,990)	0.22 (0.16–0.31)	0.61 (0.52–0.67)	91,148,998 (58,576,614–153,950,104)	51,909 (40,020–66,992)	7,161,689 (6,078,248–9,074,283)

* Includes selected species of the families Echinostomatidae, Fasciolidae, Gymnophallidae, Heterophyidae, Nanophyetidae, Neodiplostomidae and Plagiorchiidae (depending on data availability).

# Results for the enteric protozoa are included to complete the picture for foodborne parasitic diseases but are reported in detail elsewhere [5].

Illnesses are defined as the numbers of new cases in 2010. For *Taenia solium* this is estimated from GBD 2010 [9] regional incidence data and modified as the actual number of cases of epilepsy attributed to cysticercosis. The years lived with disability component of the DALY for cysticercosis is prevalence-based, estimated from Global Burden of Disease 2010 data.

### Estimating Incidence, Cases, Sequelae, and Deaths for the 11 Parasitic Diseases

Incidence is defined as the numbers of new cases per year. The incidence of each of the parasitic diseases was estimated where possible. For cysticercosis, the burden was estimated from a proportion of the prevalent epilepsy cases, i.e. the number of actual cases of disease and is further detailed below. Those incident cases with sequelae (or diseased individuals) were assigned years of life lost (YLLs) if fatal or years lived with disability (YLDs) with a disability weight (DW) that depended on the severity of the disease. For some diseases, such as toxoplasmosis, many of the incident cases do not have sequelae (i.e. they are sub-clinical). Such cases were given a DW of 0. The proportions of incident cases resulting in death or other sequelae are detailed in S1 Table.

Systematic reviews were undertaken to estimate the incidence, sequelae and mortality due to these diseases [10–16]. Where possible, public health records describing numbers of cases presenting for treatment were reviewed. These data were only available for some diseases in some countries. In others surveillance data were used (for example laboratory data on sero-conversion rates in the population).

For congenital toxoplasmosis (CT) a systematic search of 9 major databases for published and unpublished sources was undertaken and through this material direct contact with the authors of source materials was established. Searches were country-specific. To be included, studies had to report on the incidence of CT, on positivity to *Toxoplasma*-specific IgM in infants and pregnant women (including seroconversion results) or on positivity to *Toxoplasma*-specific IgG in the general population. Various modelling techniques were used, depending on the country-specific data available, to estimate the CT incidence and burden in each country. Reports of children born with CT, IgM serology of infants and pregnant women and age-stratified sero-prevalence in women and the general population combined with fertility rates of specific age groups were used to directly estimate the incidence of CT. Alternatively the data were used to input into models that were able to generate CT incidences from IgM sero positive rates in children or pregnant women or from the IgG sero conversion rates in women combined with age specific fertility rates. These data were then synthesized into an estimate of the global incidence of CT and of the global burden of CT in disability-adjusted life years (DALYs). Further details of the methodology, inclusion criteria, all the source material, PRISMA statement and the modelling techniques used are available directly in the systematic review of the global burden of congenital toxoplasmosis or the associated on line supplementary information, both accessible through [15]. Data on sero-prevalence were also used to estimate the incidence of acquired toxoplasmosis. Thus changes in seroprevalance between age of T and T+1 can be used to estimate incidence. Details are given in S1 Table.

Incidence estimates and clinical sequelae, for diseases caused by foodborne trematodes were mainly based on the results of two review articles [16,17] (S1 Table). We also imputed incidence rates for countries without reported national prevalence, but with reports of at least one autochthonous human infection, by using a hierarchical random-effects models and incidence information from other countries as input data [18]. In highly endemic zones adult subjects either maintain the parasites acquired when young or can be newly infected as the consequence of inhabiting a zone of high infection risk. This suggests that in those areas, the majority of infected adults should be chronically infected. However, acute lesions by repetitive infections are frequently superimposed on chronic disease [19]. Therefore, it is reasonable to assume that these overlapping series of repeat infections results in life-long sequelae. Thus the incidence of trematode infection was estimated from the numbers of new cases in each age cohort.

To estimate the incidence of alveolar echinococcosis (AE), due to infection with the larval stage of *Echinococcus multilocularis*, literature searches were undertaken in any relevant databases that could be accessed. These data sources were synthesized to obtain estimates of the incidences of AE in countries where *E*. *multilocularis* was known to be endemic. Further details of the strategy to obtain the data together with the methodology to estimate incidences from the data are described in [11] and S1 Table. For cystic echinococcosis (CE), due to infection with the larval stage of *E*. *granulosus* the results of a systematic review [14] and other data bases were used. The sources of data used are given in detail in S2 Text.

*T*. *solium* neurocysticercosis (NCC) is known to cause epilepsy and other neurological sequelae [12]. A meta-analysis revealed that brain lesions due to neurocysticercosis are present in approximately 29.0% (95% UI 22.9%–35.5%) of people with epilepsy in populations living in *T*. *solium* endemic areas in settings with poor sanitation and pig management practices and where pork is consumed [13]. Consequently, the incidence, prevalence, mortality and burden of disease due to epilepsy (including both idiopathic and secondary) used in the Global Burden of Disease Study 2010 (GBD 2010) [9,20–22] were used to estimate the burden of epilepsy-associated NCC. Further details, including assumptions with regard to the populations at risk, are detailed in S1 Table. The estimates of the populations at risk are detailed in S1 Data. Once the population at risk was known, 29% of the burden of epilepsy from GBD 2010 was applied to that population to estimate the burden of epilepsy attributable to NCC. Although NCC can show many other neurological and psychiatric symptoms [12], due to the absence of available consistent data on these other sequelae only the burden of NCC-associated epilepsy was estimated in this study.

Data on the global prevalence of human ascariosis stratified by age, gender and country were provided by the Institute for Health Metrics and Evaluation [9]. Based on these data and according to the methodology further explained in S1 Table and using the life expectancy of the parasite (approximately 1 year), the equivalent incident cases were estimated from the prevalence data. The sequelae proposed in GBD 2010 [20], were used in our study.

To assess the global incidence and clinical effects of human trichinellosis, outbreak reports were analyzed. Searches of six international databases yielded 494 reports, of which 261 were selected for data extraction after applying strict relevance and reliability criteria. From 1986 through 2009, there were 65,818 cases and 42 deaths reported from 41 countries. The apparent annual incidence of and mortality caused by trichinellosis was calculated by dividing the average number of cases and deaths in this 24 year period by the 1997 mid-year population. Due to the important variability in reporting of the disease, the apparent incidence and mortality rates per billion persons per year were adjusted to account for under-reporting of the cases due to under-ascertainment, medical misclassification and/or absence of effective surveillance systems. The data analysis focused on incidence, age and sex of patients, major clinical aspects including sequelae, and meat sources of infection. Full details of the search criteria, data sources, and analysis are described in [10]. The global burden of trichinellosis was subsequently estimated as described elsewhere [23].

### Disability Weights, Sequelae Duration, and Case Fatality

Disease models were developed for each of the 11 parasitic diseases to assign DWs and duration of non-fatal cases and for estimating case fatality ratios. Details of the disease models for each parasitic disease are provided in S1 Table. Where possible, DWs for outcomes and sequelae described in the GBD 2010 [20] were assigned to non-fatal parasitic diseases. DWs for individual conditions are specified in S1 Table.

### Calculation of Disability Adjusted Life Years (DALYs)

YLDs were estimated from the number of incident cases, multiplied by the DW and estimated durations of the respected sequelae. YLLs were estimated from the number of deaths and the age at death. In the case of NCC, the methodology was varied due to the nature of available data. Thus we assigned a proportion of the disease burden reported for epilepsy in GBD 2010 [9] to NCC based on the proportion of the total population that was estimated to be at risk in *T*. *solium* endemic areas as described above. So in the case of NCC we used prevalence based YLDs. However, in the absence of evidence of strong temporal trends in incidence, this is a reasonable approximation for incidence based YLDs. The normative life table used for calculating YLLs was based on the projected frontier life expectancy for 2050, with a life expectancy at birth of 92 years [24]. No age weighting or discounting was undertaken in line with recent practices [25]. DALYs are calculated by adding the adjusted number of YLDs and the number of YLLs:

YLD = Number of incident cases x Duration until remission or death x Disability Weight

YLL = Number of deaths x Residual life expectancy at the age of death

Further details of methodology to calculate DALYs are given in the companion paper in this collection on computational methods [26].

### Proportion of Burden That Is Foodborne

Fishborne trematodes and *Trichinella* spp. were assumed to be 100% foodborne based on the nature of their life cycle. In addition *Fasciola* spp. were assumed to be 100% foodborne, although there may be small opportunities for water borne transmission [16,27]. *T*. *solium* cysticercosis was assumed to be 100% foodborne, but indirectly. In other words, the *T*. *solium* life cycle cannot persist without foodborne transmission of the parasite between pigs and humans. Humans become infected by the adult stage of *T*. *solium* by eating pork, resulting in intestinal taeniosis. However, individuals who have *T*. *solium* taeniosis infect themselves or others by eggs excreted in their feces which are then ingested, often through food contamination, resulting in cysticercosis. In the complete absence of pork consumption, there would be no *T*. *solium* taeniosis and hence no cysticercosis. To estimate the proportion of the other parasitic diseases that were transmitted by food, structured expert elicitations were undertaken [28].

It can also be argued that congenital toxoplasmosis is a vertically transmitted disease rather than foodborne. However, public health measures are largely undertaken to prevent maternal (i.e., horizontal) infection which will, as a consequence, reduce the risk of fetal infection. There is relatively little evidence that treatment to prevent vertical transmission (such as antiprotozoal treatment of acutely infected pregnant women) is effective in reducing disease burden [29]. Thus it was considered as a horizontally transmitted infection to the mother, although the burden of disease is suffered mostly by the fetus, following subsequent vertical transmission. Accordingly the proportion of foodborne disease, suffered by the fetus, is the proportion of the horizontal transmission to susceptible women that occurs through food.

### Data Analysis

The FERG used an analytical approach to addressing data gaps and to estimate cases, proportion of cases afflicted with the defined sequelae, deaths, and DALYs. We defined the burden of a specific foodborne parasite as that resulting from various health states, including death, that are causally related to its transmission through food, and which may become manifest at different time scales and of different durations. We used a probabilistic approach to model the probabilities of death or the presence and duration of the various health statuses. The United Nations country-level population data for 2010 using the 2012 World Population Prospects Revision were used in all calculations which followed disease-specific computational disease models defined by incidence rates and probability parameters, each with a distribution [24]. As default, we used a log-Normal random effects model to impute missing country-level incidence data, using subregion as random effect or cluster variable. Uncertainty around input parameters was propagated using Monte Carlo simulations; 10,000 values were sampled from each input parameter to calculate 10,000 estimates of cases, deaths or DALYs. The 2.5th and 97.5th percentile of each set of the 10,000 estimates yielded a 95% UI, with the 50^th^ percentile yielding the median. The computational methodology we used is fully described in [26].

### Availability of Data

**Fig 1:**
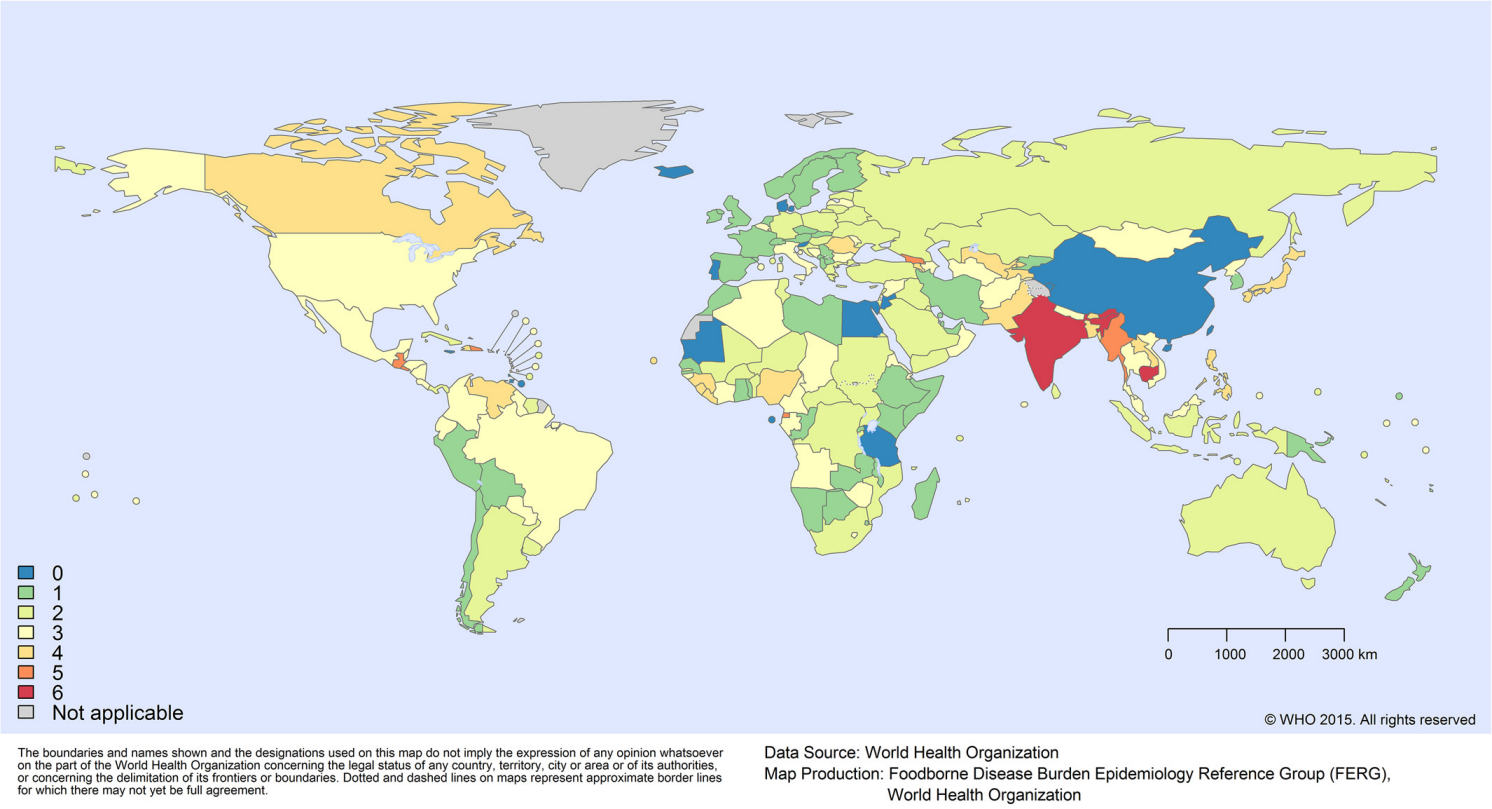
Missing data by country: number of hazards for which no data were available.

Of the 12 PDTF hazards (including congenital and acquired *Toxoplasma gondii* as separate entities), 2 hazards did not need imputation. For epilepsy due to *Taenia solium*, we applied GBD 2010 burden envelopes [9]. For trichinellosis, we applied the regional estimates generated by Devleesschauwer et al. [23]. For the 10 remaining hazards, the total number of countries with missing data ranged from 5 to 90 (out of 194 included countries). Among the 194 included countries, the number of hazards for which no data were available ranged from 0 to 6 (out of 10 hazards). For the five most populous countries in the world, the number of hazards with no data was 0 (China), 6 (India), 3 (United States), 2 (Indonesia), and 3 (Brazil). Fig 1 shows the number of data gaps per country. Availability of data is given in detail at the country level in S2 Table.

## Results

The estimated numbers of incident cases of each of the parasitic diseases are given in Table 1. The parasitic diseases with the largest total number of symptomatic incident cases and symptomatic incident cases attributable to contaminated food in 2010 are acquired toxoplasmosis and ascariosis. The incidence in 2010 of each parasitic disease per 100,000 population by region are given in Table 2. Also of note were the relatively few cases of human trichinellosis with a global estimate of just 4400 cases and 4 deaths in 2010.

**Table 2:** Median rate per 100,000 of foodborne illnesses, deaths and Disability Adjusted Life Years (DALYs) by region, with 95% uncertainty intervals.

PATHOGEN	AFR			AMR			EMR			EUR			SEAR			WPR			GLOBAL		
	ILLNESSES (95% UI)	DEATHS (95% UI)	DALYs (95% UI)	ILLNESSES (95% UI)	DEATHS (95% UI)	DALYs (95% UI)	ILLNESSES (95% UI)	DEATHS (95% UI)	DALYs (95% UI)	ILLNESSES (95% UI)	DEATHS (95% UI)	DALYs (95% UI)	ILLNESSES (95% UI)	DEATHS (95% UI)	DALYs (95% UI)	ILLNESSES (95% UI)	DEATHS (95% UI)	DALYs (95% UI)	ILLNESSES (95% UI)	DEATHS (95% UI)	DALYs (95% UI)
Enteric protozoa^#^	1,995 (549–5,866)	0.2 (0.07–0.7)	21 (6–62)	733 (222–2,077)	0.009 (0.003–0.03)	1 (0.5–3)	1,989 (560–5,118)	0.07 (0.01–0.3)	7 (2–28)	77 (21–181)	0.003 (0–0.009)	0.2 (0.04–0.7)	584 (133–1,946)	0.1 (0.03–0.4)	11 (3–36)	737 (124–2,566)	0.005 (0–0.03)	1 (0.2–4)	976 (520–1,752)	0.08 (0.04–0.2)	7 (3–15)
*Cryptosporidium* spp.^#^	205 (35–813)	0.2 (0.04–0.4)	13 (3–37)	114 (32–355)	0.007 (0.002–0.02)	0.6 (0.2–2)	346 (52–1,287)	0.04 (0.004–0.2)	4 (0.4–20)	21 (4–70)	0.003 (0–0.009)	0.2 (0.03–0.6)	78 (10–474)	0.09 (0.01–0.4)	6 (0.9–29)	32 (2–170)	0.003 (0–0.03)	0.3 (0.02–3)	125 (57–269)	0.05 (0.02–0.1)	4 (2–11)
*Entamoeba* spp.^#^	796 (98–3,868)	0.05 (0.009–0.4)	5 (0.9–39)	212 (16–1,209)	0.001 (0–0.009)	0.3 (0.03–1)	737 (79–3,110)	0.02 (0.002–0.2)	2 (0.3–14)	0 (0–0)	0 (0–0)	0 (0–0)	256 (27–1,188)	0.03 (0.004–0.2)	3 (0.3–17)	229 (0–1,598)	0.001 (0–0.003)	0.3 (0–1)	407 (149–997)	0.02 (0.007–0.08)	2 (0.7–7)
*Giardia* spp.^#^	809 (172–2,574)	0 (0–0)	0.8 (0.2–3)	309 (62–1,249)	0 (0–0)	0.3 (0.05–1)	670 (133–2,193)	0 (0–0)	0.6 (0.1–2)	54 (16–123)	0 (0–0)	0.03 (0.009–0.1)	159 (16–903)	0 (0–0)	0.1 (0.01–0.9)	354 (8–1,519)	0 (0–0)	0.3 (0.005–1)	410 (188–828)	0 (0–0)	0.4 (0.2–0.8)
Invasive infectious disease	230 (133–387)	0.03 (0.01–0.05)	21 (11–36)	160 (92–263)	0.009 (0.004–0.02)	15 (9–26)	196 (119–295)	0.02 (0.009–0.04)	19 (11–30)	119 (80–189)	0.005 (0.002–0.01)	8 (5–14)	137 (56–245)	0.006 (0.002–0.01)	10 (4–19)	117 (65–177)	0.005 (0.002–0.01)	8 (4–13)	149 (108–217)	0.01 (0.005–0.02)	12 (8–18)
*Toxoplasma gondii*, congenital	2 (0.8–4)	0.03 (0.01–0.05)	8 (4–15)	1 (0.7–2)	0.009 (0.004–0.02)	5 (3–9)	1 (0.7–3)	0.02 (0.009–0.04)	7 (4–13)	0.3 (0.2–0.7)	0.005 (0.002–0.01)	2 (1–3)	0.4 (0.1–0.9)	0.006 (0.002–0.01)	2 (0.8–5)	0.3 (0.2–0.7)	0.005 (0.002–0.01)	2 (1–4)	0.7 (0.5–1)	0.01 (0.005–0.02)	4 (2–6)
*Toxoplasma gondii*, acquired	229 (132–386)	0 (0–0)	12 (6–22)	159 (92–261)	0 (0–0)	10 (5–17)	195 (118–292)	0 (0–0)	11 (6–18)	119 (79–188)	0 (0–0)	6 (4–10)	137 (55–244)	0 (0–0)	8 (3–15)	116 (65–176)	0 (0–0)	6 (3–10)	149 (107–216)	0 (0–0)	8 (5–13)
Cestodes	15 (11–37)	2 (1–3)	177 (131–247)	4 (3–7)	0.1 (0.08–0.2)	20 (15–26)	0.7 (0.3–18)	0.009 (0.002–0.2)	0.8 (0.2–17)	1 (0.5–2)	0.04 (0.02–0.2)	2 (1–10)	9 (7–13)	0.4 (0.3–0.5)	38 (28–50)	4 (3–6)	0.6 (0.2–1)	41 (23–60)	6 (5–11)	0.5 (0.4–0.7)	46 (35–60)
*Echinococcus granulosus*	0.7 (0.2–23)	0.007 (0.001–0.2)	0.6 (0.2–18)	0.3 (0.1–3)	0.004 (0–0.04)	0.3 (0.1–3)	0.7 (0.3–18)	0.009 (0.002–0.2)	0.7 (0.2–17)	0.8 (0.4–2)	0.009 (0.003–0.03)	0.8 (0.3–2)	0.8 (0.2–2)	0.008 (0.002–0.03)	0.6 (0.2–2)	0.3 (0.09–0.7)	0.004 (0–0.01)	0.3 (0.08–0.8)	0.6 (0.4–5)	0.007 (0.002–0.06)	0.6 (0.2–5)
*Echinococcus multilocularis*	0 (0–0)	0 (0–0)	0 (0–0)	0 (0–0)	0 (0–0)	0 (0–0)	0 (0–0.002)	0 (0–0.001)	0.01 (0.004–0.05)	0.07 (0.03–0.4)	0.03 (0.01–0.2)	1 (0.4–8)	0 (0–0)	0 (0–0)	0.006 (0–0.03)	0.4 (0–0.8)	0.4 (0–0.8)	16 (0–34)	0.1 (0.01–0.2)	0.1 (0.004–0.2)	5 (0.1–9)
*Taenia solium*	14 (11–19)	2 (1–3)	175 (129–241)	3 (3–4)	0.1 (0.08–0.1)	19 (15–25)	0 (0–0)	0 (0–0)	0 (0–0)	0 (0–0)	0.002 (0.001–0.003)	0.2 (0.1–0.4)	8 (6–11)	0.4 (0.3–0.5)	37 (28–50)	4 (3–5)	0.2 (0.1–0.3)	24 (18–32)	5 (4–7)	0.4 (0.3–0.5)	41 (31–52)
Nematodes	170 (68–288)	0.02 (0.005–0.07)	9 (4–17)	130 (60–793)	0.01 (0.004–0.04)	7 (3–57)	200 (72–282)	0.02 (0.004–0.06)	10 (4–15)	8 (4–12)	0.002 (0–0.006)	0.6 (0.3–1)	255 (88–461)	0.01 (0.001–0.08)	12 (4–23)	213 (57–358)	0.01 (0.001–0.06)	10 (3–20)	179 (121–334)	0.01 (0.006–0.04)	9 (6–19)
*Ascaris* spp.	170 (68–288)	0.02 (0.005–0.07)	9 (4–17)	130 (60–793)	0.01 (0.003–0.04)	7 (3–57)	200 (72–282)	0.02 (0.004–0.06)	10 (4–15)	8 (4–11)	0.002 (0–0.006)	0.6 (0.3–1)	255 (88–461)	0.01 (0.001–0.08)	12 (4–23)	213 (57–358)	0.01 (0.001–0.06)	10 (3–20)	178 (120–334)	0.01 (0.006–0.04)	9 (6–19)
*Trichinella* spp.	0 (0–0.001)	0 (0–0)	0.001 (0–0.002)	0.06 (0.04–0.07)	0 (0–0)	0.009 (0.005–0.01)	0.002 (0–0.003)	0 (0–0)	0 (0–0)	0.4 (0.3–0.5)	0 (0–0)	0.04 (0.02–0.07)	0.002 (0–0.004)	0 (0–0)	0 (0–0.001)	0.01 (0.004–0.02)	0 (0–0)	0.004 (0.001–0.007)	0.06 (0.04–0.09)	0 (0–0)	0.008 (0.004–0.01)
Trematodes	0.006 (0.002–0.02)	0 (0–0)	0.04 (0.01–0.1)	1 (0.8–2)	0 (0–0.001)	9 (7–13)	0.6 (0.4–0.9)	0 (0–0)	5 (3–7)	0.06 (0.04–0.1)	0 (0–0)	0.4 (0.3–0.8)	0.7 (0.5–1)	0.06 (0.05–0.08)	8 (6–10)	11 (8–14)	0.4 (0.3–0.4)	97 (78–120)	3 (2–4)	0.1 (0.09–0.1)	29 (24–36)
*Clonorchis sinensis*	0 (0–0)	0 (0–0)	0 (0–0)	0 (0–0)	0 (0–0)	0 (0–0)	0 (0–0)	0 (0–0)	0 (0–0)	0 (0–0)	0 (0–0)	0.01 (0.008–0.01)	0.004 (0.001–0.01)	0 (0–0.001)	0.04 (0.01–0.1)	2 (1–2)	0.3 (0.3–0.4)	29 (24–35)	0.5 (0.3–0.7)	0.08 (0.07–0.1)	8 (6–9)
*Fasciola spp*.	0.003 (0–0.008)	0 (0–0)	0.02 (0.007–0.06)	0.5 (0.3–0.9)	0 (0–0)	4 (2–7)	0.6 (0.4–0.8)	0 (0–0)	5 (3–7)	0.006 (0.002–0.02)	0 (0–0)	0.06 (0.02–0.2)	0.006 (0.002–0.02)	0 (0–0)	0.05 (0.02–0.1)	0.09 (0.01–0.8)	0 (0–0)	0.8 (0.1–7)	0.2 (0.1–0.4)	0 (0–0)	1 (0.8–3)
Intestinal flukes*	0 (0–0.003)	0 (0–0)	0.006 (0.002–0.02)	0.009 (0.003–0.03)	0 (0–0)	0.08 (0.02–0.3)	0.009 (0.003–0.03)	0 (0–0)	0.07 (0.03–0.2)	0.006 (0.003–0.01)	0 (0–0)	0.05 (0.02–0.1)	0.02 (0.006–0.05)	0 (0–0)	0.1 (0.05–0.4)	1 (0.8–1)	0 (0–0)	8 (6–11)	0.3 (0.2–0.4)	0 (0–0)	2 (2–3)
*Opisthorchis* spp.	0 (0–0)	0 (0–0)	0 (0–0)	0 (0–0)	0 (0–0)	0 (0–0)	0 (0–0)	0 (0–0)	0 (0–0)	0.05 (0.04–0.08)	0 (0–0)	0.3 (0.2–0.5)	0.6 (0.4–0.9)	0.06 (0.05–0.07)	8 (6–10)	0.2 (0.2–0.3)	0.02 (0.02–0.03)	3 (2–3)	0.2 (0.2–0.3)	0.02 (0.02–0.03)	3 (2–3)
*Paragonimus* spp.	0.002 (0–0.007)	0 (0–0)	0.02 (0.005–0.05)	0.6 (0.4–0.8)	0 (0–0.001)	5 (3–7)	0.002 (0–0.006)	0 (0–0)	0.02 (0.006–0.05)	0.001 (0–0.004)	0 (0–0)	0.009 (0.003–0.03)	0.008 (0.002–0.04)	0 (0–0)	0.06 (0.02–0.3)	7 (5–10)	0.01 (0.008–0.02)	55 (39–76)	2 (1–3)	0.004 (0.002–0.005)	15 (11–21)
**TOTAL (excluding enteric protozoa)**	418 (277–644)	2 (1–3)	208 (159–283)	293 (195–1,035)	0.1 (0.1–0.2)	51 (41–112)	398 (253–535)	0.05 (0.03–0.3)	35 (25–58)	128 (89–199)	0.05 (0.03–0.2)	11 (8–24)	404 (220–649)	0.5 (0.4–0.6)	69 (54–89)	346 (188–512)	1 (0.5–1)	156 (127–193)	337 (265–553)	0.7 (0.5–0.9)	96 (82–122)
**TOTAL**	2,428 (934–6,426)	2 (2–3)	232 (176–317)	1,060 (507–2,790)	0.1 (0.1–0.2)	53 (42–113)	2,390 (933–5,535)	0.1 (0.05–0.4)	44 (30–76)	210 (136–328)	0.05 (0.03–0.2)	12 (8–24)	1,007 (461–2,491)	0.6 (0.5–1)	80 (61–114)	1,089 (429–3,088)	1 (0.6–1)	158 (128–195)	1,325 (851–2,237)	0.8 (0.6–1)	104 (88–132)

* Includes selected species of the families Echinostomatidae, Fasciolidae, Gymnophallidae, Heterophyidae, Nanophyetidae, Neodiplostomidae and Plagiorchiidae (depending on data availability).

# Enteric protozoa are included to complete the picture for foodborne parasitic diseases but are reported in detail elsewhere [5].

Illnesses are defined as the numbers of new cases in 2010. For *Taenia solium* this is estimated from GBD 2010 [9] regional incidence data and modified as the actual number of cases of epilepsy attributed to cysticercosis. The YLD component of the DALY for cysticercosis is prevalence-based, estimated from GBD 2010 data.

AFR, African Region; AMR, Region of the Americas; EMR, Eastern Mediterranean Region; EUR, European Region; SEAR, South-East Asian Region; WPR, Western Pacific Region.

The number of DALYs associated with each parasite and the proportion of DALYs that were foodborne in 2010 are given in Table 1. In 2010 the burdens estimated to be caused by cysticercosis were 2.79 million (95% UI 2.14–3.61 million) DALYs. Foodborne trematodosis resulted in 2.02 million (95% UI 1.65–2.48 million) DALYs. Toxoplasmosis had a burden (congenital and acquired combined) of 1.68 million (95% UI 1.24–2.45 million) DALYs, with ascariosis also resulting in 1.32 million (95% UI 1.18–2.70 million) DALYs. Echinococcosis (alveolar and cystic combined), had a burden of approximately 871,000 DALYs (CE 184,000, 95% [UI 88,100–1.59 million] DALYs; AE 688,000, 95% [UI 409,000–1.1 million] DALYs). This gives a 2010 global burden of these 11 parasitic diseases of 8.78 million (95% UI 7.62–12.5 million) DALYs, of which 6.64 million (95% UI 5.61–8.41 million) DALYs were estimated to be foodborne. Contaminated food may be responsible for 48% (95% UI 38%–56%) of incident cases and approximately 76% (95% UI 65%–81%) of DALYs (Table 1). Stillbirths were excluded, although in the case of congenital toxoplasmosis, if counted as deaths as an alternative scenario, this would result in 4,470 (95% UI 969–12,400) additional deaths and hence an addition of approximately 411,000 (95% UI 89,100–1.14 million) YLLs. Of these approximately 2,180 (95% UI 470–6,090) deaths and 200,000 (95% UI 43,200–560,000) YLLs would be foodborne.

**Fig 2:**
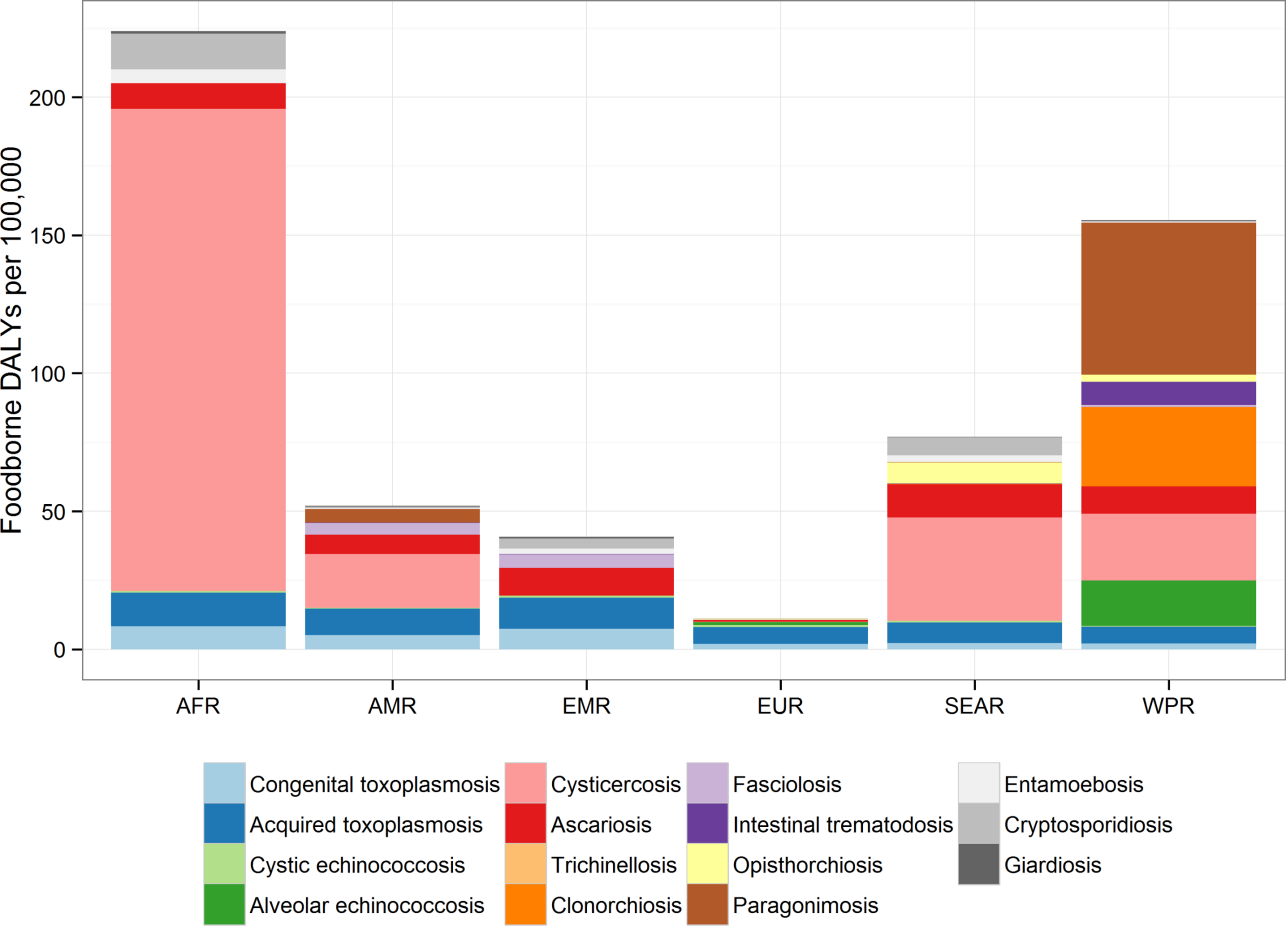
Contribution of each parasite to foodborne Disability Adjusted Life Years in regions: the relative contribution to the DALY incidence by each agent for each of the regions. This includes enteric protozoa to complete the picture on foodborne parasitic diseases. However, details about enteric protozoa are reported in the research article by Kirk et al on foodborne enteric pathogens [5].

The 2010 incidence rates of foodborne-attributable symptomatic disease, death and DALYs caused by each of these parasitic diseases per 100,000 by region are given in Table 2. The largest global incidence rate of DALYs was found in the Western Pacific and African regions with 156 (95% UI 127–193) and 208 (95% UI 159–283) DALYs per 100,000, respectively, whereas the lowest was found in the European region with 11 (95% UI 8–24) DALYs per 100,000. However, the relative importance of the different parasitic infections varied across regions and this is clearly illustrated in Fig 2. For example, the burden of opisthorchiosis is largely confined to South East Asian subregion D, whilst cysticercosis is rarely seen in either Eastern Mediterranean or European regions.

**Fig 3:**
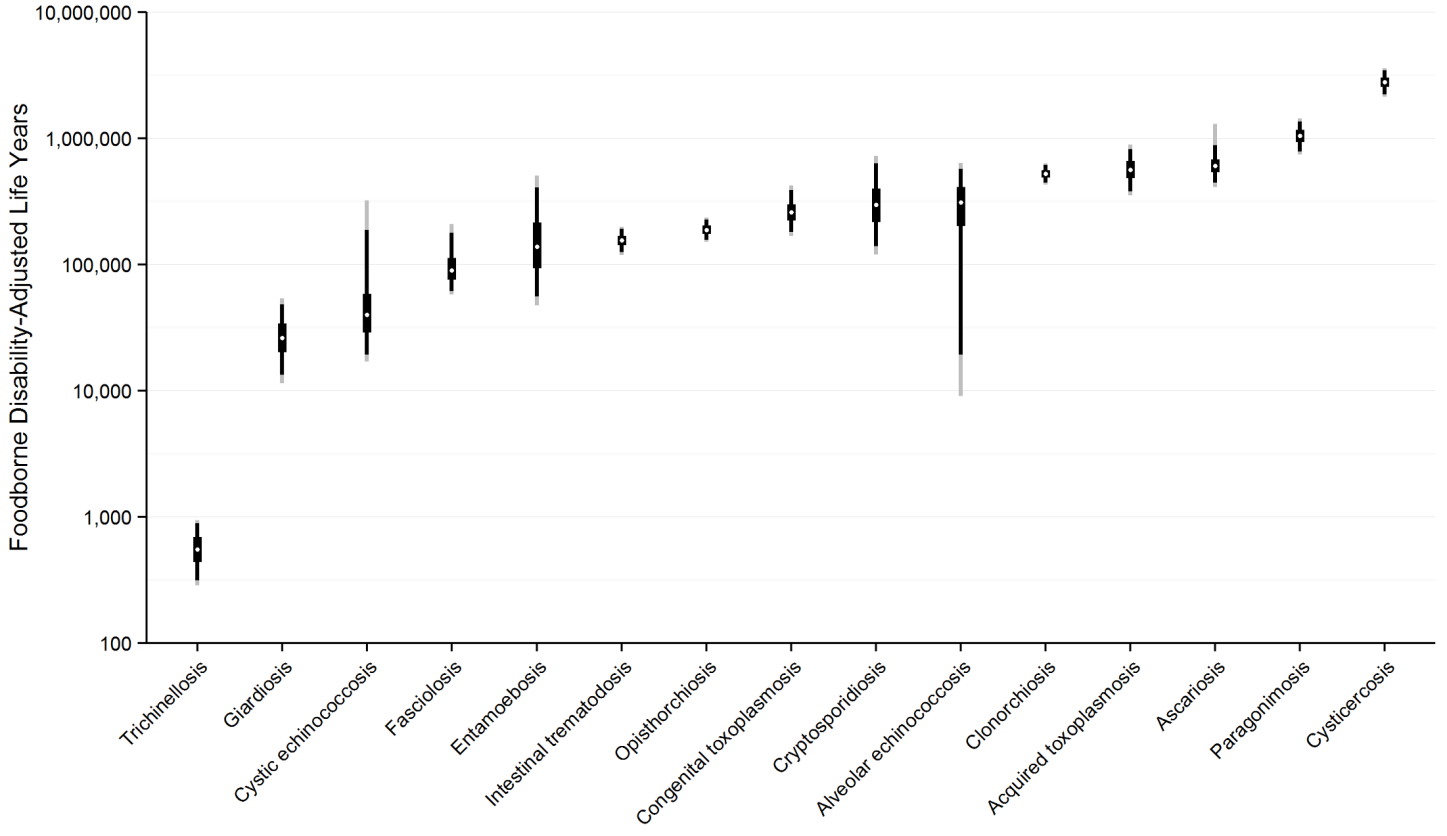
Worldwide foodborne Disability Adjusted Life Years by parasite: Disability Adjusted Life Years for each parasite acquired from contaminated food ranked from lowest to highest with 95% Uncertainty Intervals, 2010. This includes enteric protozoa to complete the picture on foodborne parasitic diseases. However, details about enteric protozoa are reported in the research article by Kirk et al on foodborne enteric pathogens [5].

**Fig 4:**
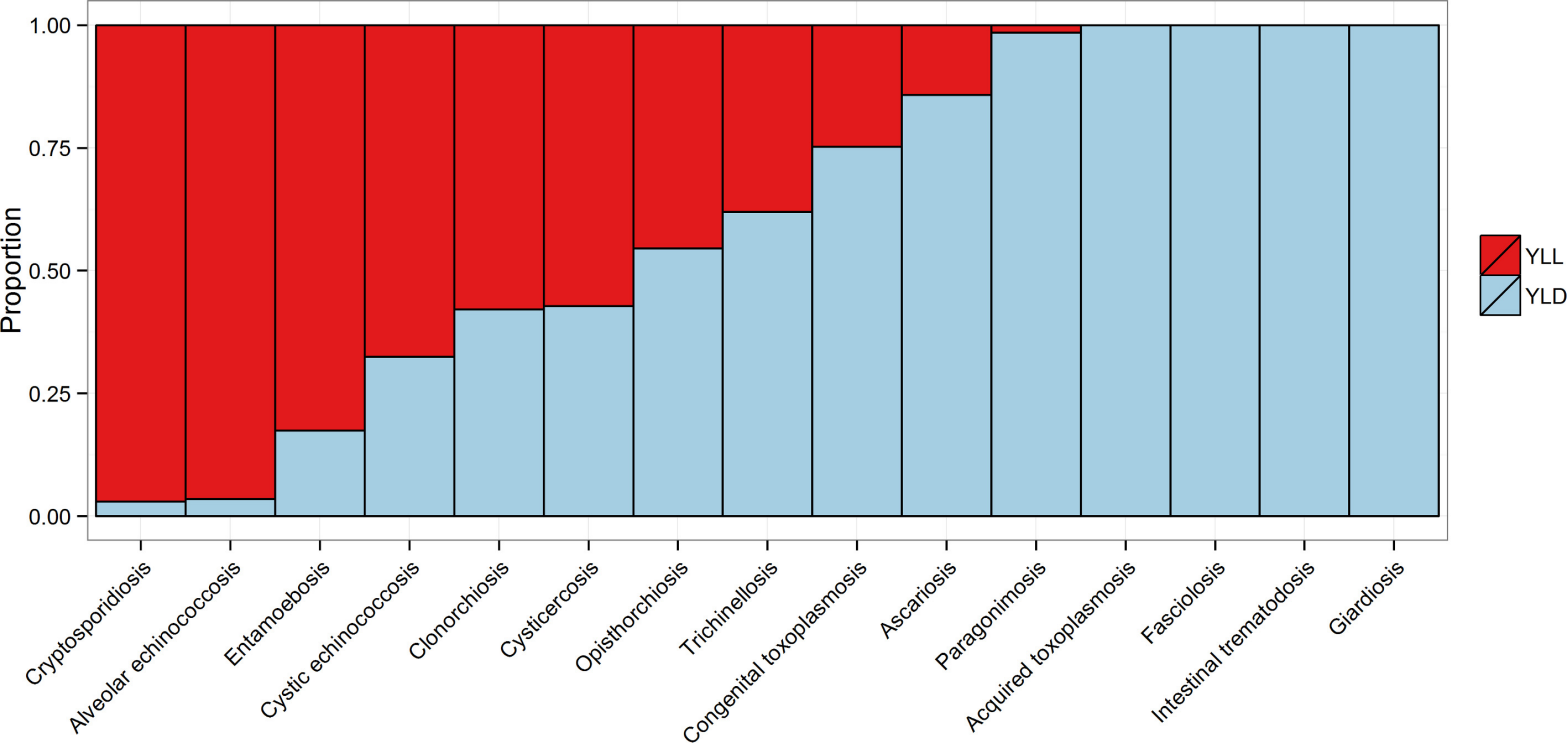
The relative proportion of the burden of each of the foodborne parasitic diseases contributed by YLLs and YLDs.

The absolute and relative foodborne burdens of these parasitic diseases, including the three enteric protozoa, are illustrated in Fig 3. The relative proportion of the burden of each of the foodborne parasitic diseases contributed by YLLs and YLDs is illustrated in Fig 4.

## Discussion

In this study, we estimate for the first time the disease burden imposed by foodborne parasites. The results highlight the significant burden in low- and middle-income countries where cycles of parasitic infection are highly specific to food sources. In addition to those detailed here, a further 357 million cases, 33,900 deaths and 2.94 million DALYs are due to enteric protozoa of which 67.2 million cases, 5,560 deaths and 492,000 DALYs are attributable to foodborne transmission (see [5] and Tables 1 and 2). These complete the picture for the foodborne parasitic diseases given available data.

We used the best evidence available combined with the natural history of the disease to obtain estimates of the incidence, mortality and sequelae of each parasitic disease. Several of the diseases were included in GBD 2010 [9]. In a number of cases our estimates for the global burden of disease differ quite substantially from those of GBD 2010. The estimate for echinococcosis (which combined AE and CE in one estimate) in GBD 2010 is 144,000 DALYs [9]. This is less than a fifth of our combined median estimate of 871,000 DALYs. This discrepancy probably reflects different methodologies between the two studies. GBD 2010 relied heavily on vital records for mortality attributed to these diseases, whereas we used a natural history of disease approach. Our choice of approach was strongly influenced by the chronic nature of these diseases and that often only prevalence data were available. In addition, these diseases often have their highest impact in low income countries where vital records are likely to be poor and hospital treatment unavailable. Our estimates for the global burden of CE would arguably be more consistent with an earlier estimate [30] if there had been no substantial methodological differences. The earlier report suggested a median estimate of 285,000 DALYs assuming no under reporting, rising to 1 million DALYs where under-reporting was assumed. The earlier report also used DWs ranging from 0.200 to 0.809, depending on the severity of the disease. In the present study we used a maximum DW of 0.221, and this was only applied to the relatively small number of neurological cases. Echinococcosis of the abdominal organs, the most common presentation of the disease, had a DW of 0.123 for treatment seeking cases in the present study. The former study also undertook age weighting and discounting that we decided not to incorporate into this study. In addition different life tables were used. Our use of DWs was guided by GBD 2010 and the results of a systematic review of the clinical manifestations of CE [14]. However, a median estimate in excess of 188,000 cases of CE per year, with the possibility of up to 1.77 million new cases, indicates a substantial burden. With a low case-fatality rate, the burden in terms of DALYs is highly dependent on the DW and duration of illness. Neither of these is defined with certainty. The lack of defined DWs specific for the differing sequelae of CE must be seen as a major data gap. When arriving at the estimates for AE, it was assumed that in excess of 90% of cases outside of Europe would be fatal. This assumption was supported by survival analyses confirming that in the absence of aggressive treatment of this disease, including chemotherapy, most cases die [31,32]. Our results suggest it is possible that the global burden of AE may be somewhat higher than that of CE, which may at first sight seem surprising as there are many more cases of CE globally and the parasite has a more cosmopolitan distribution. Although we have a median estimate of CE incidence that is ten times higher than the median estimate of AE incidence, the high case fatality ratio of AE, results in the loss of 37 DALYs per case compared to 0.98 DALYs for each case of CE. Thus the global burden of AE was driven by the large number of YLLs. For CE it was driven by the YLDs.

Our estimates for cysticercosis were higher than those of GBD 2010 [9]. This is because we assigned a substantial proportion of the epilepsy burden to cysticercosis based on the results of a systematic review [13]. Furthermore, a subsequent systematic review has largely confirmed our findings in terms of the fraction of epilepsy attributable to NCC [33]. However, our results are not inconsistent with GBD 2010 [9] because we have allocated some of the burden from epilepsy to a specific aetiological agent. Nevertheless, the present estimate in this report may still underestimate the burden of cysticercosis, as there are other important clinical symptomatology associated with NCC, such as chronic headache, hydrocephalus, stroke and depressive disorders [12]. Better estimates of the role that cysticercosis plays in stroke and depressive disorders globally could considerably increase its burden estimates since these conditions are ranked third and eleventh, respectively, in the GBD 2010 [9] estimates. Furthermore, it is also unclear how GBD 2010 arrived at their estimates for cysticercosis. If, for example, it was assumed that cysticercosis-related epilepsy can only be attributed in individuals who are serologically positive for cysticercosis this would lead to substantive underestimates. A large proportion of cases of epilepsy attributed to cysticercosis, as shown by imaging studies, are nevertheless seronegative. For example Montano et al [34] describe 15 cases of epilepsy aetiologically confirmed as NCC, but only 7 of these were seropositive.

Likewise, the estimates for the burden of foodborne trematode infections in this study may also represent underestimates. Our estimates were based on the results of an earlier study, which used estimation methods that were conservative [16]. Often, population-level information on human foodborne trematode infections were completely lacking from areas where the parasites are endemic, as indicated by substantial rates of animal infections and human food habits that suggest transmission to humans to be likely. We tried to correct for this lack of data by imputing incidence rates for all countries with at least one autochthonous human infection reported in the reviewed literature. Nevertheless, and in line with the original study [16], only very conservative estimates from the imputation were accepted in an attempt to avoid inflating the burden estimates for human foodborne trematode infections based on unclear evidence.

Some diseases such as toxoplasmosis were not estimated in GBD 2010 and will inevitably have been included in other syndromes. For example, congenital defects in GBD 2010 will have incorporated the DALYs for congenital toxoplasmosis that we have estimated in the present study.

With the exception of NCC, we have used an incidence approach to estimating the YLDs. This is where the YLD part of the DALY was estimated from number of incident cases per year multiplied by the DW and duration. This contrasts with the GBD 2010 approach which used a prevalence approach to YLDs where YLDs were estimated by number of prevalent cases multiplied by the DW. For acute disease in generally stable epidemiological situations (i.e. no considerable shifts in the epidemiological key indicators of prevalence, incidence, duration, severity, remission and mortality) and settings with more or less stable population size, these alternative approaches result in few differences [35]. But for chronic diseases in populations that are rapidly increasing, the prevalence approach may underestimate the numbers of YLDs. Parasitic diseases are often chronic and are often of highest incidence in low income countries with increasing populations. Many parasitic diseases have durations of many years, or in the case of congenital toxoplasmosis, the sequelae are usually lifelong. Thus, as we adopted the GBD 2010 data for epilepsy to estimate the burden of NCC, the YLDs will be prevalence based. Nearly all of the burden of NCC is in low income countries, which usually have increasing populations. Therefore the cohort at the time of infection, to which the burden is attributed in an incidence-based approach, will be larger than earlier cohorts which are still affected by NCC but are reported in the prevalence-based approach. Accepting this limitation means that the estimates for epilepsy attributed to NCC will result in a further underestimate of the burden of cysticercosis.

We have summarized the differences between the estimates for GBD 2010 and the FERG estimates for these pathogens, including the enteric protozoa in Table 3. In addition, an issue that appears common to many hazards is that GDB 2010 [9] has not published many of their search strategies, or modeling methods to deal with data deficiencies. Until these are published we will only be able to hypothesise the reasons for some of the differences in the estimates.

**Table 3:** Comparisons of the total burden of parasitic diseases (foodborne and non-foodborne) estimated by FERG and GBD 2010 [9].

Parasite	GBD	FERG	Hypothesised reasons for differences in GBD and FERG estimates
*Cryptosporidium* spp.	8,372,000 (6,473,000–10, 401,000)	2,159,331 (1,392,438–3,686,925)	Differences in DALYs estimated by GBD2010 and FERG are largely due to differences in how aetiology-specific deaths were estimated. FERG estimated aetiology-specific deaths using the methodology adopted by CHERG*[36]. GBD used a modelling based approach to estimate aetiology specific deaths, but there is no description of the GBD model available to review. GBD has not published the studies included, their search strategy, or modelling methods; until these are published it is not possible to completely compare GBD and FERG estimates.
*Entamoeba histolytica* (Ameobiosis)	2,237,000 (1,728,000–2,832,000)	515,904 (222,446–1,552,466)	
*Giardia* spp.	Not estimated	171,100 (115,777–257,315)	
*Toxoplasma gondii*	Not estimated	1,684,414 (1,236,005–2,452,060)	Assumed to be included in congenital diseases and non-specific communicable diseases in GBD
*Echinococcus granulosus*	152,000 (60,000–359,000)	183,573 (88,082–1,590,846)	GBD used vital records which are often missing in low resource countries. FERG used a natural history approach based on surveillance data. GBD used prevalence based YLDs, which will underestimate burden for a chronic disease like echinococcosis. Methods for imputation of missing data were different. GBD has not published their modeling methods for missing data.
*Echinococcus multilocularis*		687,823 (409,190–1,106,320)	
*Taenia solium*	514,000 (398,000–650,000)	2,788,426 (2,137,613–3,606,582)	GBD used vital records relying on a diagnosis of cysticercosis. FERG assigned a substantial proportion of the epilepsy envelope to cysticercosis in resource poor, pork consuming communities based on evidence from a systematic review and meta-analysis. GBD has not published their modeling methods for missing data.
*Ascaris lumbricoides*	1,315,000 (713,000–2,349,000)	1,317,535 (1,182,187–2,700,572)	Only subtle differences as FERG and GBD used the same source data, but FERG estimated incidence based YLDs whereas GBD used prevalence based YLDs.
*Trichinella* spp.	Not estimated	550 (285–934)	
Foodborne Trematodes	1,875,000 (708,000–4,837,000)	2,024,592 (1,652,243–2,483,514)	Only subtle differences as FERG and GBD used the same source data, but FERG estimated incidence based YLDs whereas GBD used prevalence based YLDs.

* Child Health Epidemiology Reference Group of the World Health Organization and UNICEF

FERG, Foodborne Disease Burden Epidemiology Reference Group; GBD 2010, Global Burden of Disease Study 2010; YLD, years lived with disability.

The limitations in this study are similar to others in this collection. There were often substantial data gaps that had to be filled by imputation and suffer from the uncertainties that surround such models. Excluding stillbirths is consistent with the approach used to estimate the burden due to enteric pathogens [5]. Congenital toxoplasmosis is the only pathogen we investigated that could result in a substantial incidence of stillbirths. However an estimate for the burden of congenital toxoplasmosis that includes stillbirths as equivalent to neonatal deaths has been reported as 1.2 million DALYs per annum [15]. In our report we have assumed that acquired toxoplasmosis usually results in a relatively mild acute illness with some cases suffering fatigue for a few months duration [37]. Although fatal cases have been recorded [38], these were assumed to be uncommon and hence zero YLLs were estimated. We have also assumed that although acquired chorioretinitis occurs following toxoplasmosis it only occurs in a small proportion of cases (see S1 Table). This results in approximately 1.15 million DALYs in 2010 from an estimated 20.7 million people having clinical disease following exposure to the pathogen for the first time. However, there is increasing evidence that acquired toxoplasmosis may result in a number of neurological or psychiatric diseases such as schizophrenia and epilepsy. In GBD 2010 these diseases resulted in 15.0 million and 17.4 million DALYs respectively. From two meta-analyses [39, 40] and a large cross-sectional study conducted in China [41], it is possible to estimate that the population attributable fraction of schizophrenia associated with seropositivity to toxoplasmosis is approximately 9%, which on a crude level could account for approximately 1.3 million additional DALYs.

There were also some notable omissions from our study. *Taenia saginata*, which causes human taeniosis and is transmitted solely from beef was not considered because the parasite produces very mild, unapparent clinical disease in affected humans which would result in a DW of close to zero and hence a very low burden of human disease. However, it is accepted that this parasite generates substantial economic damage because of meat inspection and trade regulations required in many countries to detect and remove the parasite from the food chain [42]. Likewise other cestode zoonoses, where the adult tapeworm is located in the gastrointestinal tract (e. g. *Diphyllobothrium* spp.) with few clinical signs were also not included. In contrast, trichinellosis was considered to be an important foodborne pathogen with potentially serious disease. However, this study has suggested that the global burden of trichinellosis is small. This is discussed elsewhere [23]. For reasons of resources we were not able to consider foodborne Chagas disease although it was suggested as a possible priority pathogen during the second FERG meeting [8]. However, particularly recently, the assumption that Chagas disease is primarily a vectorborne disease is being questioned [43]. For example, 70% of cases of acute Chagas disease recorded in Brazil between 2000 and 2010 were associated with food consumption [44]. As GBD 2010 made an estimate of the burden of Chagas disease of 546,000 DALYs [9] there could be a significant additional burden through foodborne transmission if these data are representative. Indeed, foodborne Chagas disease may turn out to have a higher burden then the foodborne burden of some of the pathogens we have considered such as *Trichinella* and *Giardia* spp. We were also unable to estimate the burden of foodborne cyclosporosis. This has caused outbreaks in the USA such as the multistate outbreak of 631 cases in 2013 [45]. However, the total numbers of cases over the medium to long term appears to be quite small with a median annual incidence of 0.03 cases per 100,000 [46]. Thus any contribution to the burden of disease by this pathogen is likely to be small.

A further important limitation was relying on expert elicitation for the proportion of disease that is foodborne. This was an important issue with those parasitic diseases such as ascariosis, toxoplasmosis and echinococcosis, that can have several pathways of transmission. Expert elicitation studies can result in a highly variable proportions attributed to food. However, as data on source attribution for a number of parasites were not available the structured elicitation undertaken offered a transparent way of evaluating and enumerating this uncertainty and thus represents the best available source of information [5,28]. The expert elicitation for routes of transmission estimated that approximately a median of 15% (UIs 7–27%) of *Giardia* infections were transmitted via contaminated food. This is was higher than we expected for this enteric protozoan. For example, Scanlan et al 2011 [47], suggested that 7% of *Giardia* infections acquired in the USA were of foodborne origin. However, in contrast a recent 40-year summary of outbreaks of giardiosis reported to the United States Centers for Disease Control and Prevention identified that 16% of 242 outbreaks were the true result of foodborne transmission [48]. Both these studies suggested that the proportion of foodborne giardiosis is within the 95% uncertainty limits of our study. Furthermore, a recent report by the Food and Agriculture Organization (FAO) and WHO presented a multi-criteria ranking of 24 (groups of) foodborne parasites, and concluded that giardiosis was the 11^th^ most important foodborne parasite [49,50] with fresh produce likely to be the vehicle of transmission. This indicates that it is accepted this parasite has a foodborne transmission route and puts our estimates in this context.

Likewise, the use of imputation where no data is available will lead to inaccuracies, and those countries where we used imputation can be seen in S2 Table. In addition, we used epilepsy and *Ascaris* prevalence data from GBD 2010 to inform our estimates of cysticercosis and foodborne ascariosis respectively. Therefore the accuracy of our estimates will be limited to the accuracy of the GBD 2010 data from which is was derived.

*T*. *gondii* is globally distributed with a high proportion of the world population estimated to be seropositive. *A*. *lumbricoides* is the most frequently encountered human helminth although the burden is confined to low and middle income countries. However, a number of diseases had very high burdens limited to distinct geographical populations. Most of the global burden of AE is in China, and mainly on the Tibetan plateau [11]. In this highland region there are specific factors that promote transmission between wildlife, dogs and humans that are not present in other endemic areas. This results in large numbers of human cases in certain communities [51]. Such unique epidemiological conditions are not present elsewhere, even where the parasite is endemic. *T*. *solium* transmission can only be maintained where pork is consumed, pigs are left roaming and where there is poor sanitation. Thus it is largely absent from upper income countries and from communities where pork is not consumed, such as countries in the Middle East. Sporadic cases are occasionally reported and these are often linked to the employment of immigrants who originate from endemic countries and hence transmit the infection through poor hygienic practices [52]. Foodborne trematodes also have a limited distribution, but they cause a high burden of disease in the at risk populations such as South East Asia. Trematodes have complex life cycles which include various species of molluscs. This limits their distribution to specific regions where suitable life cycle hosts are endemic, which may be adapted to specific climatic and hydrological conditions [53]. The human disease is further limited to populations that are likely to consume raw fish or undercooked aquatic vegetables that are the source of transmission. Consequently, although we are reporting the global burden of these parasitic diseases, this is often borne almost completely by relatively small populations in limited geographical areas. Therefore, in such communities, these diseases have a major impact on the health of the population.

A recent report by the FAO and WHO presented a ranking of foodborne parasites, based on multicriteria analysis [49,50]. In our study, we present data on the foodborne disease burden for 13 parasites included in the FAO/WHO report. Comparing the results of the ranking from the FAO/WHO model with the results of the present study, the parasites selected by FERG had the highest rank orders in the FAO/WHO report (i.e., ranking from 1 to 14), only *Trypanosoma cruzi* at rank 11 and *Cyclospora cayetanensis* at rank 13 were not assessed by FERG. *T*. *solium* was ranked 1 by both approaches and *T*. *gondii* 3 by FERG and 4 by FAO/WHO. There were, however, also remarkable differences in the ranking of the individual parasites. *Paragonimus* spp. was ranked 2 by FERG, but only 14 in the FAO/WHO report and *E*. *granulosus* 12 by FERG but 2 by FAO/WHO. The foodborne disease burden of *E*. *multilocularis* was considerably higher than the foodborne burden of *E*. *granulosus* (310,000 vs. 40,000 DALYs), but nevertheless was ranked lower at 3 by FAO/WHO. The disease burden of intestinal flukes was 9 by FERG. This was higher than the 22 ranking of heterophyidae by FAO/WHO. FAO and WHO used 9 criteria for ranking, of which 6 were health related criteria and 3 non-health criteria. This weighting of the different criteria may be responsible for the FAO/WHO having a different ranking order of various parasites. For example *E*. *granulosus* has a global distribution, which is a relatively important measure in the FAO/WHO ranking. In contrast, *E*. *multilocularis* is only found in the northern hemisphere.

In summary our results provide important information for those developing and implementing food safety policy. We have shown that some parasites such as foodborne trematodes, *T*. *gondii* and *T*. *solium* produce considerable burdens of preventable parasitic diseases, whilst others such as *Trichinella* spp. result in a low burden. There are also data gaps which might be filled in future local, country or regional studies. Such studies will make further contributions to food safety.

## Supporting Information

S1 Text Glossary of terms and acronyms. (DOC)

S2 Text Sources of data used to estimate the incidence of cystic echinococcosis by country. (DOC)

S1 Table Incidence, clinical outcomes, duration, disability weights, mortality, age and sex distributions of 11 parasitic diseases potentially transmitted through food. (XLSX)

S2 Table Availability of data for estimating the global and regional burden of foodborne parasitic disease. No data indicates imputation was undertaken (see main text).
(XLSX)

S1 Data Estimated population at risk for cysticercosis by country. (XLSX)

## Abbreviations

AE: alveolar echinococcosis
CE: cystic echinococcosis
CT: congenital toxoplasmosis
DALYs: Disability Adjusted Life Years
DW: disability weight
FAO: the Food and Agricultural Organization
FERG: Foodborne Disease Burden Epidemiology Reference Group
GBD 2010: Global Burden of Disease Study 2010
NCC: neurocysticercosis
PDTF: Parasitic Diseases Task Force
UI: Uncertainty intervals
WHO: World Health Organization
YLL: years of life lost
YLD: years lived with disability
