# Supplementary material for: World Health Organization Estimates of the Global and Regional Disease Burden of 11 Foodborne Parasitic Diseases, 2010: A Data Synthesis
Source: PLoS Med. 2015 Dec 3;12(12):e1001920. doi: 10.1371/journal.pmed.1001920 (PMC4668834; doi:10.1371/journal.pmed.1001920)
Supplement: S1 Text — (DOC) [file pmed.1001920.s001.doc]

**GLOSSARY**

*Foodborne disease*

A foodborne disease (FBD) can be defined as a disease commonly transmitted through ingested food. FBDs comprise a broad group of illnesses, and may be caused by microbial pathogens, parasites, chemical contaminants and biotoxins.

*Burden of disease*

In the context of this Initiative, the term “burden of disease” follows the principles of the Global Burden of Disease Study, and includes the quantification of morbidity, all disabling complications and mortality in a single summary measure (DALY).

*DALY (disability-adjusted life year)*

A health gap measure that combines the years of life lost due to premature death (YLL) and the years lived with disability (YLD) from a disease or condition, for varying degrees of severity, making time itself the common metric for death and disability. One DALY equates to one year of healthy life lost.

*Food*

According to the Codex Alimentarius Commission, “food means any substance, whether processed, semi-processed or raw, which is intended for human consumption, and includes drink, chewing gum and any substance which has been used in the manufacture, preparation or treatment of food but does not include cosmetics or tobacco or substances used only as drugs”(Murray *et al.*, 2013). The definition includes all bottled drinks.

*Source attribution*

Source attribution (SA) is the partitioning of the human burden of a particular disease to specific sources. With regards to foodborne diseases, SA can be conducted at various points along the food distribution chain, from the animal reservoir to the point of consumption.

**ABBREVIATIONS**

AE Alveolar Echinococcosis

AFR African Region

AMR Region of the Americas

UI Uncertainty Interval

CHERG Child Health Epidemiology Reference Group

CE Cystic Echinococcosis

CT Congenital Toxoplasmosis

CTF Computational Task Force of FERG

DALY Disability-Adjusted Life Year

EMR Eastern Mediterranean Region

EUR European Region

FAO Food and Agriculture Organization of the United Nations

FERG Foodborne Disease Burden Epidemiology Reference Group

GBD Global Burden of Disease

NCC Neurocysticercosis

PDTF Parasitic Diseases Task Force of FERG

SEAR South-East Asian Region

WHO World Health Organization

WPR Western Pacific Region

YLD Years Lived with Disability

YLL Years of Life Lost

WORLD HEALTH ORGANIZATION REGIONS

**African Region:**

Algeria, Angola, Benin, Botswana, Burkina Faso, Burundi, Cameroon, Cape Verde, Central African Republic, Chad, Comoros, Congo, Côte d’Ivoire, Democratic Republic of the Congo, Equatorial Guinea, Eritrea, Ethiopia, Gabon, Gambia, Ghana, Guinea, Guinea-Bissau, Kenya, Lesotho, Liberia, Madagascar, Malawi, Mali, Mauritania, Mauritius, Mozambique, Namibia, Niger, Nigeria, Rwanda, Sao Tome and Principe, Senegal, Seychelles, Sierra Leone, South Africa, Swaziland, Togo, Uganda, United Republic of Tanzania, Zambia, Zimbabwe.

**Region of the Americas:**

Antigua and Barbuda, Argentina, Bahamas, Barbados, Belize, Bolivia, Brazil, Canada, Chile, Colombia, Costa Rica, Cuba, Dominica, Dominican Republic, Ecuador, El Salvador, Grenada, Guatemala, Guyana, Haiti, Honduras, Jamaica, Mexico, Nicaragua, Panama, Paraguay, Peru, Saint Kitts and Nevis, Saint Lucia, Saint Vincent and the Grenadines, Suriname, Trinidad and Tobago, United States of America, Uruguay, Venezuela.

**South-East Asia Region:**

Bangladesh, Bhutan, Democratic People’s Republic of Korea, India, Indonesia, Maldives, Myanmar, Nepal, Sri Lanka, Thailand, Timor-Leste.

**European Region:**

Albania, Andorra, Armenia, Austria, Azerbaijan, Belarus, Belgium, Bosnia and Herzegovina, Bulgaria, Croatia, Cyprus, Czech Republic, Denmark, Estonia, Finland, France, Georgia, Germany, Greece, Hungary, Iceland, Ireland, Israel, Italy, Kazakhstan, Kyrgyzstan, Latvia, Lithuania, Luxembourg, Malta, Monaco, Montenegro, Netherlands, Norway, Poland, Portugal, Republic of Moldova, Romania, Russian Federation, San Marino, Serbia, Slovakia, Slovenia, Spain, Sweden, Switzerland, Tajikistan, The former Yugoslav Republic of Macedonia, Turkey, Turkmenistan, Ukraine, United Kingdom, Uzbekistan.

**Eastern Mediterranean Region:**

Afghanistan, Bahrain, Djibouti, Egypt, Iran, Iraq, Jordan, Kuwait, Lebanon, Libyan Arab Jamahiriya, Morocco, Oman, Pakistan, Qatar, Saudi Arabia, Somalia, South Sudan, Sudan, Syrian Arab Republic, Tunisia, United Arab Emirates, Yemen.

**Western Pacific Region:**

Australia, Brunei Darussalam, Cambodia, China, Cook Islands, Fiji, Japan, Kiribati, Lao People’s Democratic Republic, Malaysia, Marshall Islands, Micronesia, Mongolia, Nauru, New Zealand, Niue, Palau, Papua New Guinea, Philippines, Republic of Korea, Samoa, Singapore, Solomon Islands, Tonga, Tuvalu, Vanuatu, Viet Nam.
