# Supplementary material for: World Health Organization Estimates of the Global and Regional Disease Burden of 11 Foodborne Parasitic Diseases, 2010: A Data Synthesis
Source: PLoS Med. 2015 Dec 3;12(12):e1001920. doi: 10.1371/journal.pmed.1001920 (PMC4668834; doi:10.1371/journal.pmed.1001920)
Supplement: S2 Text — (DOC) [file pmed.1001920.s002.doc]

Supporting Information 2

Sources of data used to estimate the incidence of cystic echinococcosis by country. When the country is believed to be not endemic for *Echinococcus granulosus* this is stated. Otherwise countries with no data sources the incidence was imputed as described in the main text.

Region Page

AFR 2

AMR 6

EMR 9

EUR 11

SEAR 17

WPR 18

AFR Region

|  | No of cases | Cases/year | Incidence/100,000 years | Year | Source |
| --- | --- | --- | --- | --- | --- |
| Algeria | 3337 | 556 | 1.65 | 2004-2009 | Anon (2004-2009) |
| Angola |  |  |  |  |  |
| Benin |  |  |  |  |  |
| Botswana | 80 | 27 |  |  | Hájek et al (2004) |
| Burkina Faso |  |  |  |  |  |
| Burundi |  |  |  |  |  |
| Cameroon |  |  |  |  |  |
| Cape Verde |  | 0 |  |  | Not endemic |
| Central African Republic |  |  |  |  |  |
| Chad |  |  |  |  |  |
| Cote d'Ivoire |  |  |  |  |  |
| Democratic Republic of Congo |  |  |  |  |  |
| Equitorial Guinea |  |  |  |  |  |
| Eritrea |  |  |  |  |  |
| Ethiopia |  |  | 7.65 (median) |  | Dawit and Shishay, 2014; Fuller and Fuller, 1981; Kassa, 2012; Kebede et al., 2010; Mulatu et al., 2013; Teffera et al., 2013 |
| Gabon |  |  |  |  |  |
| Gambia |  |  |  |  |  |
| Ghana |  |  |  |  |  |
| Guinea |  |  |  |  |  |
| Guinea-Bissau |  |  |  |  |  |
| Kenya |  |  | 3.2 (estimated from references) |  | Addy et al., 2012; Casulli et al., 2009; Cooney et al., 2004; Gathura and Kamiya, 1990; Macpherson et al., 1986; Mbaya et al., 2014; Mutwiri et al., 2014 |
| Lesotho |  |  |  |  |  |
| Liberia |  |  |  |  |  |
| Madagascar |  |  | 0 |  | Not endemic |
| Malawi |  |  |  |  |  |
| Mali |  |  |  |  |  |
| Mauritania | 24 |  | 1.2 |  | Salem et al., 2011 |
| Mauritius |  |  | 0 |  | Not endemic |
| Mozambique |  |  |  |  |  |
| Namibia |  |  |  |  |  |
| Niger |  |  |  |  |  |
| Nigeria |  |  |  |  |  |
| Rwanda |  |  |  |  |  |
| Sao Tome and Principe |  |  | 0 |  | Not endemic |
| Senegal |  |  |  |  |  |
| Seychelles |  |  | 0 |  | Not endemic |
| Sierra leone |  |  |  |  |  |
| South Africa |  |  |  |  |  |
| Swaziland |  |  |  |  |  |
| Togo |  |  |  |  |  |
| Uganda |  |  |  |  |  |
| United Republic of Tanzania |  |  | 0.1 (minimum if most cases occur in the north) |  | Ernest et al., 2010 |
| Zambia |  |  |  |  | Banda (2013) |
| Zimbabwe |  |  |  |  |  |

**References**

Addy, F., Alakonya, A., Wamae, N., Magambo, J., Mbae, C., Mulinge, E., Zeyhle, E., Wassermann, M., Kern, P., Romig, T., 2012. Prevalence and diversity of cystic echinococcosis in livestock in Maasailand, Kenya. Parasitol. Res. 111, 2289–2294. doi:10.1007/s00436-012-3082-8

Anon (2004-2009) R.E.M. Relevé Epidémioloique Annuel. Vols XV-XX Institute National de Santé Publique, Algerie. Available from: <http://www.ands.dz/insp/>

Banda, F., 2013. Prevalence and risk factors of cystic echinococcosis in cattle and humans in western province of Zambia (MSc). University of Zambia, Lusaka.

Casulli, A., Zeyhle, E., Brunetti, E., Pozio, E., Meroni, V., Genco, F., C, F., 2009. Molecular evidence of the camel strain (G6 genotype) of Echinococcus granulosus in humans from Turkana, Kenya. Trans. R. Soc. Trop. Med. Hyg. (in press) doi:10.1016/j.trstmh.2009.08.001.

Cooney, R.M., Flanagan, K.P., Zehyle, E., 2004. Review of surgical management of cystic hydatid disease in a resource limited setting: Turkana, Kenya. Eur. J. Gastroenterol. Hepatol. 16, 1233–1236.

Dawit, G., Shishay, K., 2014. Epidemiology, Public Health Impact and Control Methods of the Most Neglected Parasite Diseases in Ethiopia: A Review. World J. Med. Sci. 10, 92–102.

Ernest, E., Nonga, H.E., Kynsieri, N., Cleaveland, S., 2010. A retrospective survey of human hydatidosis based on hospital records during the period 1990-2003 in Ngorongoro, Tanzania. Zoonoses Public Health 57, e124–129. doi:10.1111/j.1863-2378.2009.01297.x

Fuller, G.K., Fuller, D.C., 1981. Hydatid disease in Ethiopia: clinical survey with some immunodiagnostic test results. Am. J. Trop. Med. Hyg. 30, 645–652.

Gathura, P.B., Kamiya, M., 1990. Echinococcosis in Kenya: transmission characteristics, incidence and control measures. Jpn. J. Vet. Res. 38, 107–116.

Kassa, S.A., 2012. Cystic hydatidosis in Ethiopia: a review. Sci. J. Crop Sci. 1, 1–8. doi:10.14196/sjcs.v1i1.60

Kebede, N., Mitiku, A., Tilahun, G., 2010. Retrospective survey of human hydatidosis in Bahir Dar, north-western Ethiopia. East. Mediterr. Health J. Rev. Santé Méditerranée Orient. Al-Majallah Al-Ṣiḥḥīyah Li-Sharq Al-Mutawassiṭ 16, 937–941.

Macpherson, C.N., Wachira, T.M., Zeyhle, E., Romig, T., Macpherson, C., 1986. Hydatid disease: research and control in Turkana, IV. The pilot control programme. Trans. R. Soc. Trop. Med. Hyg. 80, 196–200.

Mbaya, H., Magambo, J., Njenga, S., Zeyhle, E., Mbae, C., Mulinge, E., Wassermann, M., Kern, P., Romig, T., 2014. Echinococcus spp. in central Kenya: a different story. Parasitol. Res. 113, 3789–3794. doi:10.1007/s00436-014-4045-z

Hájek M, Novák K, Chudácek Z., 2004[Echinococcus cysts (hydatids)] Chirurgické oddelení Vojenské nemocnice v Plzni. Rozhledy v Chirurgii : Mesicnik Ceskoslovenske Chirurgicke Spolecnosti [2004, 83(6):227-235]

Mulatu, M., Mekonnen, B., Tassew, H., Kumar, A., 2013. Bovine Hydatidosis in Eastern Part of Ethiopia. Momona Ethiop. J. Sci. 5, 107–114. doi:10.4314/mejs.v5i1.

Mutwiri, T., Magambo, J., Zeyhle, E., Mkoji, G.M., Wamae, C.N., Mulinge, E., Mbae, C., Wassermann, H., Kern, P., Romig, T., 2014. Molecular characterisation of echinococcus granulosus species/strains in human infections from Turkana, Kenya. East Afr. Med. J. 90, 235–240. doi:10.4314/eamj.v90i7.

Salem, C.O.A., Schneegans, F., Chollet, J., Jemli, M. et, 2011. Epidemiological Studies on Echinococcosis and Characterization of Human and Livestock Hydatid Cysts in Mauritania. Iran. J. Parasitol. 6, 49–57.

Teffera, E., Kassa, S., Ali, A., 2013. Patterns of Cardiothoracic and vascular surgical admissions at a tertiary University hospital Addis Ababa, Ethiopia. East Cent Afr J Surg 18, 121–126.

AMR

|  | No of cases | Cases/year | Incidence/100,000 years | Year | Source |
| --- | --- | --- | --- | --- | --- |
| Argentina | 2291 | 406 | 1.96^1^ | 2005-2010 | Ministerio de Sallud; Presidencia de la Nacion 2012 |
| Bahamas |  |  | 0 |  | Not Endemic |
| Barbados |  |  | 0 |  |  |
| Belize |  |  |  |  |  |
| Bolivia |  |  |  |  |  |
| Brazil |  |  | 0.065^2^ |  | Farias et al., 2004; Rue and L, 2008 |
| Canada | 108 | 26 | 0.07 | 2001-2005 | Gilbert et al., 2010 |
| Chile |  | 1009 | 5.7 | 2001-2009 | Martínez G, 2011 |
| Columbia |  |  |  |  |  |
| Cuba |  |  | 0 |  | Not endemic |
| Dominica |  |  | 0 |  | Not endemic |
| Dominican republic |  |  |  |  |  |
| Ecuador |  |  |  |  |  |
| El Salvador |  |  |  |  |  |
| Grenada |  |  | 0 |  | Not Endemic |
| Guatemala |  |  |  |  |  |
| Guyana |  |  |  |  |  |
| Haiti |  |  |  |  |  |
| Honduras |  |  |  |  |  |
| Jamaica |  |  | 0 |  | Not endemic |
| Mexico |  |  |  |  |  |
| Nicaragua |  |  |  |  |  |
| Panama |  |  |  |  |  |
| Paraguay |  |  |  |  |  |
| Peru |  |  | 10 |  | Moro et al., 2011 |
| St Kitts and Nevis |  |  | 0 |  | Not endemic |
| St Lucia |  |  | 0 |  | Not endemic |
| St Vincent and the Grenadines |  |  | 0 |  | Not endemic |
| Surinam |  |  | 0 |  | Not endemic |
| Trinidad and Tobago |  |  | 0 |  | Not endemic |
| USA | 41^3^ deaths (=2050 cases) | 121 | 0.04 | 1990-2007 | Bristow et al., 2012 |
| Uruguay |  | 367 | 6.5^4^ | 1993 |  |
| Venezuala |  |  |  |  |  |

^1^Adjusted for underreporting. Official figures c 0.95/100,000 per year

^2^Nationwide incidence estimated from data of Rio Grande do Sul State, which is the only endemic region in the country.

^3^Only fatalities reported. Case numbers estimated assuming a 2% case fatality rate.

^4^ Incidence assumed to have reduced since 1997

**References**

Bristow, B.N., Lee, S., Shafir, S., Sorvillo, F., 2012. Human Echinococcosis Mortality in the United States, 1990–2007. PLoS Negl Trop Dis 6, e1524. doi:10.1371/journal.pntd.0001524

Farias, L.N., Malgor, R., Cassaravilla, C., Bragança, C., de la Rue, M.L., 2004. Echinococcosis in southern Brazil: efforts toward implementation of a control program in Santana do Livramento, Rio Grande do Sul. Rev. Inst. Med. Trop. São Paulo 46, 153–156. doi:/S0036-46652004000300006

Gilbert, N.L., Dare, O.K., Libman, M.D., Muchaal, P.K., Ogden, N.H., 2010. Hospitalization for trichinellosis and echinococcosis in Canada, 2001-2005: the tip of the iceberg? Can. J. Public Health Rev. Can. Santé Publique 101, 337–340.

Martínez G, P., 2011. Hidatidosis humana: antecedentes generales y situación epidemiológica en Chile, 2001-2009. Rev. Chil. Infectol. 28, 585–591. doi:10.4067/S0716-10182011000700013

Ministerio de Sallud; Presidencia de la Nacion 2012. Enfermedades Infecciosas Hidatidosis Diagnóstico de Hidatidosis Guia Para El Equipo De Salud. Dirección de Epidemiología - Ministerio de Salud de la Nación, Buonas Aries Agentina, 47pp. Avialable from: <http://www.msal.gov.ar/zoonosis/images/stories/info-equipos-de-salud/pdf/hidatidosis-guia-medica.pdf>

Morelli, A., Maurin, L., Agulla, J., Sambran, Y., Islas, W., Sapia, M., Vignolo, J., 1996. Hidatidosis en el Uruguay. Prevallencia Quirurgica Nacional 1993. Comision Nacional Honoraria de Lucha Contra la Hidatidosis, Montevideo.

Moro, P.L., Budke, C.M., Schantz, P.M., Vasquez, J., Santivañez, S.J., Villavicencio, J., 2011. Economic Impact of Cystic Echinococcosis in Peru. PLoS Negl Trop Dis 5, e1179. doi:10.1371/journal.pntd.0001179

Rue, de L., L, M., 2008. Cystic echinococcosis in southern Brazil. Rev. Inst. Med. Trop. São Paulo 50, 53–56. doi:10.1590/S0036-46652008000100012

EMR

|  | No of cases | Cases/year | Incidence/100,000 years | Year | Source |
| --- | --- | --- | --- | --- | --- |
| Afghanistan |  |  |  |  |  |
| Bahrain |  |  |  |  |  |
| Djibouti |  |  |  |  |  |
| Egypt |  |  | 0.79 |  | Kandeel et al., 2004 |
| Iran |  |  | 1.24 |  | Fasihi Harandi et al., 2012 |
| Iraq |  |  |  |  |  |
| Jordan |  |  | 2.9 |  | Al-Qaoud et al., 2003; Kamhawi, 1995 |
| Kuwait |  |  | 0.75 |  | Shalabi et al., 2002 |

| Libyan Arab Jamahiriya |  |  |  |  |  |
| --- | --- | --- | --- | --- | --- |
| Morocco | 3360 | 1680 | 5.2 | 2007-2008 | Derfoufi et al., 2012 |
| Oman |  |  |  |  |  |
| Pakistan |  |  |  |  |  |
| Qatar |  |  |  |  |  |
| Saudi Arabia |  |  |  |  |  |
| Somalia |  |  |  |  |  |
| Sudan |  |  |  |  |  |
| Syrian Arab republic |  |  |  |  |  |
| Tunisia |  |  | 12.6 |  | Chahed et al., 2010 |
| UAE |  |  |  |  |  |
| Yemen |  |  | 3.9 |  | Al-Shibani et al., 2012 |

**References**

Al-Qaoud, K.M., Craig, P.S., Abdel-Hafez, S.K., 2003. Retrospective surgical incidence and case distribution of cystic echinococcosis in Jordan between 1994 and 2000. Acta Trop. 87, 207–214. doi:10.1016/S0001-706X(03)00022-6

Al-Shibani, L.A.N., Al-Eryani, S.M.A., Azazy, A.A., Al-Mekhlafi, A.M., 2012. Cases of hydatidosis in patients referred to Governmental hospitals for cyst removal in Sana’a City, Republic of Yemen. Trop. Biomed. 29, 18–23.

Chahed, M.K., Bellali, H., Touinsi, H., Cherif, R., Ben Safta, Z., Essoussi, M., Kilani, T., 2010. [Distribution of surgical hydatidosis in Tunisia, results of 2001-2005 study and trends between 1977 and 2005]. Arch. Inst. Pasteur Tunis 87, 43–52.

Derfoufi, O., Akwa, E.N., Elmaataoui, A., Miss, E., Esselmani, H., Lyagoubi, M., Aoufi, S., 2012. Epidemiological profile of cystic echinococcosis in Morocco from 1980 to 2008. Ann. Biol. Clin. (Paris) 70, 457–461. doi:10.1684/abc.2012.0727

Fasihi Harandi, M., Budke, C.M., Rostami, S., 2012. The monetary burden of cystic echinococcosis in iran. PLoS Negl. Trop. Dis. 6, e1915. doi:10.1371/journal.pntd.0001915

Kamhawi, S., 1995. A retrospective study of human cystic echinococcosis in Jordan. Ann. Trop. Med. Parasitol. 89, 409–414.

Kandeel, A., Ahmed, E.S., Helmy, H., El Setouhy, M., Craig, P.S., Ramzy, R.M.R., 2004. A retrospective hospital study of human cystic echinococcosis in Egypt. East. Mediterr. Health J. Rev. Santé Méditerranée Orient. Al-Majallah Al-Ṣiḥḥīyah Li-Sharq Al-Mutawassiṭ 10, 349–357.

Shalabi, R.I., Ayed, A.K., Amin, M., 2002. 15 Years in surgical management of pulmonary hydatidosis. Ann. Thorac. Cardiovasc. Surg. Off. J. Assoc. Thorac. Cardiovasc. Surg. Asia 8, 131–134.

EUR

|  | No of cases | Cases/year | Incidence/100,000 years | Year | Source |
| --- | --- | --- | --- | --- | --- |
| Albania | 169 | 169 | 5.6 | 2010 | WHO |
| Armenia | 234  1470 | 234  210 | 7.8 | 2010  1997-2003 | WHO  Khachatryan, and Davisyants, (2011) |
| Austria | 104 (total)  25 (Authotchonous) | 3 | 0.04 | 1978-2008 | Schneider et al., 2010 |
| Azerbaijan | 339 | 85 | 0.93 | 2003, 2007-2008, 2010 | WHO |
| Belarus | 62 | 10 | 0.1 | 2003-2004, 2006-2007, 2009-2010 | WHO |
| Belgium |  | 35 | 0.3 | 2009 | European hospital morbidity data bases |
| Boznia | 238 | 34 | 0.7 | 2003-2010 | WHO |
| Bulgaria | 3542 | 506 | 6.9 | 2003-2008, 2010 | WHO |
| Croatia | 160  86 | 23  86 | 1.9 | 2003-2010  2012 | WHO  European hospital morbidity data bases |
| Cyprus | 16  5 | 2  5 | 0.5 | 2003-2010  2011 | WHO  European hospital morbidity data bases |
| Czech Republic | 15 | 1.4 | 0.01 | 2004-2014 | WHO |
| Denmark |  | 16 | 0.3 | 2006 | European hospital morbidity data bases |
| Estonia | 8 | 1 | 0.08 | 2003-2013 | WHO |
| Finland | 9 | 1 | 0.02 | 2003-2013 | WHO |
| France | 278 | 56 | 0.09 | 2004-2006, 2008, 2010 | WHO |
| Georgia | 478 | 68 | 1.5 | 2003-2008, 2010 | WHO |
| Germany | 552 (total)  107 (authotchonous) | 42  8 | 0.05 | 2001-2013 | Robert Kochs Institute (2002-2014) |
| Greece | 128 | 21 | 0.2 | 2003-2005;2007-2010 | WHO |
| Hungary | 51 | 7 | 0.07 | 2003-2010 | WHO |
| Iceland | 0 | 0 | 0 |  | WHO |
| Ireland | 3 | 0 | 0 | 2004-2010 | WHO |
| Israel |  | 42 | 0.6 | 2000 | European hospital morbidity data bases |
| Italy | 10237 | 1000 | 1.67 | 2001-2011 | Brundu et al., 2014 |
| Kazakhstan | 2632 | 877 | 5.3 | 2003, 2007, 2008 | WHO |
| Kyrgyzstan | 1856 | 928 | 17.5 | 2011-2012 | Kyrgyz Government Report, 2013 |
| Lativia | 36 | 36 | 1.64 | 2011 | European hospital morbidity data bases |
| Lithuania | 33 | 33 | 1.5 | 2011 | European hospital morbidity data bases |
| Luxembourg | 1 | 1 | 0.2 | 2011 | European hospital morbidity data bases |
| Malta | 0 | 0 | 0 | 2004-2010 | WHO |
| Montenegro | 5 | 2 | 0.29 | 2006-2008 | WHO |
| Netherlands | 0 | 49 | 0.3 | 2011 | ECDC reports |
| Norway | 4 | 1 | 0.02 | 2003-2008, 2010 | WHO |
| Poland | 218 | 218 | 0.57 | 2011 | European hospital morbidity data bases |
| Portugal | 36 | 6 | 0.07 | 2003,2005,2007,2009-2010 | WHO |
| Republic of Molodva | 1770 | 177 | 4.9 | 2001-2010 | Lungu, 2013 |
| Romania |  | 660 | 3.3 | 1979-2010 | Moldovan et al., 2012; Neghina et al., 2011, 2010; Vlad et al., 2013 |
| Russia | 2863 | 579 | 0.4 | 2006-2010 | Russian Government Reports 2007-2011. |
| San Marino |  | 0 | 0 |  | WHO |
| Serbia | 820 | 68 | 0.92 | 1998-2010 | Bobić et al., 2012 |
| Slovakia | 27 | 5 | 0.09 | 2003-2008; 2010 | WHO |
| Slovenia | 38 | 4 | 0.2 | 2003-2011 | WHO |
| Spain | 1332 | 167 | 0.36 | 2003-2010 | WHO |
| Sweden | 95 | 16 | 0.18 | 2004-2008, 2010 | WHO |
| Switzerland | 60 | 69 | 0.91 | 2011 | European hospital morbidity data bases |
| Takjikistan |  | 1800 | 25 |  | Torgerson et al 2006 |
| The FYR of Macedonia | 116 | 19 | 0.95 | 2003-2007; 2009 | WHO |
| Turkey | 14789 | 2958 | 3.9 | 2001-2005 | Yazar et al., 2008 |
| Turkmenistan |  | 200 | 4.2 |  | Torgerson et al 2006 |
| Ukraine | 437 | 146 | 0.32 | 2003, 2007, 2010 | WHO |
| UK |  | 54 | 0.088 | 2003-2010 | European hospital morbidity data bases |
| Uzbekistan | 11874 | 3958 | 14.5 | 2000,2001, 2012 | (Hong et al., 2013; Nazirov et al., 2002) |

**References**

Bobić, B., Nikolić, A., Radivojević, S.K., Klun, I., Djurković-Djaković, O., 2012. Echinococcosis in Serbia: an issue for the 21st century? Foodborne Pathog. Dis. 9, 967–973. doi:10.1089/fpd.2012.1227

Brundu, D., Piseddu, T., Stegel, G., Masu, G., Ledda, S., Masala, G., 2014. Retrospective study of human cystic echinococcosis in Italy based on the analysis of hospital discharge records between 2001 and 2012. Acta Trop. 140C, 91–96. doi:10.1016/j.actatropica.2014.08.011

ECDC (2013) Surveillance Report 2013. Annual epidemiological report: reporting on 2011surveillance data and 2012 epidemic intelligence data. Avaialble from <http://www.ecdc.europa.eu/en/publications>

European hospital morbidity data bases <http://data.euro.who.int/hmdb/index.php>

Hong, S.-T., Jin, Y., Anvarov, K., Khadjibaev, A., Hong, S., Ahmedov, Y., Otaboev, U., 2013. Infection status of hydatid cysts in humans and sheep in Uzbekistan. Korean J. Parasitol. 51, 383–385. doi:10.3347/kjp.2013.51.3.383

Khachatryan, A. S. and Davisyants, V. A, (2011) Pathomorphology characteristics and some clinical-epidemiological patterns of echinococcosis in Armenia. Ministry of health Armenia National Instirtute of Public health in the Name of SH Avdalbekyan, Eravan, 108pp

Kyrgyz Government Report 2013. Overview of infectious diseases 28pp (In Russian) Available at: <http://www.dgsen.kg/podrazdel.php?podrazdel=36>

Lungu. V. (2013) Optimization of the Epidemiological Surveillance of Echinococcosis in the Republic of Moldova. PhD thesis, Chisinau, State University of Medicine and Pharmacy, Ministry of Health Moldova, 128pp

Moldovan, R., Neghina, A.M., Calma, C.L., Marincu, I., Neghina, R., 2012. Human cystic echinococcosis in two south-western and central-western Romanian counties: A 7-year epidemiological and clinical overview. Acta Trop. 121, 26–29. doi:10.1016/j.actatropica.2011.10.003

Neghina, R., Neghina, A.M., Marincu, I., Iacobiciu, I., 2010. Epidemiology and epizootology of cystic echinococcosis in Romania 1862-2007. Foodborne Pathog. Dis. 7, 613–618. doi:10.1089/fpd.2009.0489

Nazirov, F.G., Ilkhamov, I.L., Ambekov, N.C., 2002. Echinococcosis in Uzbekistan: types of problems and methods to improve treatment. Med. J. Uzb. 2/3, 2–5.

Neghina, R., Neghina, A.M., Marincu, I., Iacobiciu, I., 2011. Cystic echinococcosis in Romania: the pediatric approach. Vector Borne Zoonotic Dis. Larchmt. N 11, 993–999. doi:10.1089/vbz.2010.0238

Robert Koch Institute (2002-2014) Infektionsepidemiologisches Jahrbuch meldepflichtiger Krankheiten für 2001- 2013 Robert Koch-Institut, Berlin, 2002- 2014. Available at: http://www.rki.de/DE/Content/Infekt/Jahrbuch/jahrbuch_node.html

Russian Government Reports 2007-2011. The Sanitary Epidemiological Situation in the Russian Federation 2006-2010. Federal Service for Supervision in Consumer Rights Protection and Human Welfare Moscow (in Russian). Federal Center of Hygiene and Epidemiology. Available from: http://www.rospotrebnadzor.ru/files/documents/

Schneider, R., Gollackner, B., Schindl, M., Tucek, G., Auer, H., 2010. Echinococcus canadensis G7 (Pig Strain): An Underestimated Cause of Cystic Echinococcosis in Austria. Am. J. Trop. Med. Hyg. 82, 871–874. doi:10.4269/ajtmh.2010.09-0639

Torgerson, P.R., Oguljahan, B., Muminov, A.E., Karaeva, R.R., Kuttubaev, O.T., Aminjanov, M., Shaikenov, B., 2006. Present situation of cystic echinococcosis in Central Asia. Parasitol. Int. 55 Suppl, S207–212. doi:10.1016/j.parint.2005.11.032

Vlad, D.C., Neghina, A.M., Dumitrascu, V., Marincu, I., Neghina, R., Calma, C.L., 2013. Cystic echinococcosis in children and adults: a seven-year comparative study in western Romania. Foodborne Pathog. Dis. 10, 189–195. doi:10.1089/fpd.2012.1281

Yazar, S., Ozkan, A.T., Hökelek, M., Polat, E., Yilmaz, H., Ozbilge, H., Ustün, S., Koltaş, I.S., Ertek, M., Sakru, N., Alver, O., Cetinkaya, Z., Koç, Z., Demirci, M., Aktaş, H., Parsak, C.K., Ozerdem, D., Sakman, G., Cengiz, Z.T., Ozer, A., Keklik, K., Yemenici, N., Turan, M., Daştan, A., Kaya, E., Tamer, G.S., Girginkardeşler, N., Türk, M., Sinirtaş, M., Evci, C., Kiliçturgay, S., Mutlu, F., Artiş, T., 2008. [Cystic echinococcosis in Turkey from 2001-2005]. Türkiye Parazitolojii Derg. Türkiye Parazitoloji Derneği Acta Parasitol. Turc. Turk. Soc. Parasitol. 32, 208–220.

WHO centralised information system for infectious diseases <http://data.euro.who.int/cisid>

SEAR

|  | No of cases | Cases/year | Incidence/100,000 years | Year | Source |
| --- | --- | --- | --- | --- | --- |
| Bangladesh |  |  |  |  |  |
| Bhutan |  |  |  |  |  |
| DPR Korea |  |  |  |  |  |
| India |  |  | 1.84 |  | Khurana et al., 2007; Singh et al., 2014 |
| Indonesia |  |  | 0 |  | Not endemic |
| Maldives |  |  | 0 |  | Not endemic |
| Myanmar |  |  |  |  |  |
| Nepal |  |  | 0.53 |  | Devleesschauwer et al., 2014 |
| Sri Lanka |  |  |  |  |  |
| Thailand |  |  | 0 |  | Not endemic |

**References**

Devleesschauwer, B., Ale, A., Torgerson, P., Praet, N., Maertens de Noordhout, C., Pandey, B.D., Pun, S.B., Lake, R., Vercruysse, J., Joshi, D.D., Havelaar, A.H., Duchateau, L., Dorny, P., Speybroeck, N., 2014. The Burden of Parasitic Zoonoses in Nepal: A Systematic Review. PLoS Negl Trop Dis 8, e2634. doi:10.1371/journal.pntd.0002634

Khurana, S., Das, A., Malla, N., 2007. Increasing trends in seroprevalence of human hydatidosis in North India: a hospital-based study. Trop. Doct. 37, 100–102.

Singh, B.B., Dhand, N.K., Ghatak, S., Gill, J.P.S., 2014. Economic losses due to cystic echinococcosis in India: Need for urgent action to control the disease. Prev. Vet. Med. 113, 1–12. doi:10.1016/j.prevetmed.2013.09.007

WPR

|  | Cases/year | Incidence/100,000 years | Source |
| --- | --- | --- | --- |
| Australia | 50 | 0.2 | See Australia ^1^ |
| Brunei Darussalam |  | 0 | Not endemic |
| Cambodia |  | 0 | Not endemic |
| China |  | 0.95 | See China ^2^ |
| Cook islands |  | 0 | Not endemic |
| Fiji |  | 0 | Not endemic |
| Japan |  | 0 | Not endemic |
| Kiribati |  | 0 | Not endemic |
| Lao People's Democratic Republic |  | 0 | Not endemic |
| Malaysia |  | 0 | Not endemic |
| Marshall Islands |  | 0 | Not endemic |
| Micronesia |  | 0 | Not endemic |
| Mongolia |  | 6 | See Mongolia ^3^ |
| Nauru |  | 0 | Not endemic |
| New Zealand |  | 0 | Previously endemic, eliminated |
| Niue |  | 0 | Not endemic |
| Palau |  | 0 | Not endemic |
| Phillipines |  | 0 | Not endemic |
| Republic of Korea |  | 0 | Not endemic |
| Samoa |  | 0 | Not endemic |
| Singapore |  | 0 | Not endemic |
| Solomon Islands |  | 0 | Not endemic |
| Tuvalu |  | 0 | Not endemic |
| Vanuatu |  | 0 | Not endemic |

1. Australia is endemic for *Echinococcus granulosus*, especially in the widlife (Dingos and Wallabys). It is also present in many rural livestock raising areas. The disease in humans is uncommon and sporadic, and there is no public health data available to indicate numbers of human cases. Expert opinion of those working on Echinococcosis in Australia suggests there may be approximately 50 cases per year. Because the epidemiology of disease is unlike anywhere else, it would not be appropriate to model the estimates based on data from other countries in WPRO. Older data from new South Wales and Australian Capital Territory indicates a mean of 39 cases per year diagnosed between 1987-1992 (Jenkins and Power, 1996). More recently there is a review of the epidemiology of Echinococcosis in Australia (Jenkins, 2006) and recent case reports (Figtree et al., 2012, Burgess et al., 2012).

**References**

Burgess, C., Masters, I.B., Francis, P., Grimwood, K., Chang, A.B., 2012. Flexible bronchoscopy in managing a child with pulmonary hydatid disease. Pediatr. Pulmonol. 47, 1140–1142. doi:10.1002/ppul.22545

Figtree, M.C., Watts, M.R., Timmins, R., Smith, R., Hudson, B.J., 2012. Extensive cystic hydatid disease. Med. J. Aust. 196.

Jenkins, D.J., 2006. Echinococcus granulosus in Australia, widespread and doing well! Parasitol. Int. 55, S203–S206. doi:10.1016/j.parint.2005.11.031

Jenkins, D.J., Power, K., 1996. Human Hydatidosis in New South Wales and the Australian Capital Territory, 1987-1992. Med. J. Aust. 164, 18–21.

2. China. The Western part of China is highly endemic for Echionococcus granulosus. High prevalences are seen in much of the patoralists in the Tibetan plateau (Tibet Autonomus province, Western Sichuan, Qinhhai), Ningjia, Xingjiang and some other regions. Most of the data for China is from mass ultrasound studies, which estimate prevalence rather than incidence.Incidence can be estimated from the prevalence and life expectancy at the time of diagnosis assuming equilibrium. In addition, ultrasound will underestimate the prevalence by at least 10% as it can not detect cysts that are not in the abdomen, such as pulmonary cysts. Data from the following sources were used to make the estimates for China.

**References**

Chu, X., Wang, G., Feng, X., Er, X., He, J., Wen, H., 2010. [Risk factors on human cystic echinococcosis in Hobukesar Mongolian Autonomous County in Xinjiang]. Zhonghua Liu Xing Bing Xue Za Zhi Zhonghua Liuxingbingxue Zazhi 31, 297–299.

Craig, P.S., Giradoux, P., Shi, D., Bartholomot, G., Barnish, G., Delattre, P., Quere, J.P., Harraga, S., Bao, G., Wang, Y., Lu, F., Ito, A., Vuitton, D.A., 2000a. An epidemiological and ecological study on human alveolar echinococcosis transmission in Gansu, China. Acta Trop. 77, 167–177.

Craig, P.S., Giraudoux, P., Shi, D., Bartholomot, B., Barnish, G., Delattre, P., Quere, J.P., Harraga, S., Bao, G., Wang, Y., Lu, F., Ito, A., Vuitton, D.A., 2000b. An epidemiological and ecological study of human alveolar echinococcosis transmission in south Gansu, China. Acta Trop 77, 167–77.

Han, X., Wang, H., Cai, H., Ma, X., Liu, Y., Wei, B., Ito, A., Craig, P.S., 2009. [Epidemiological survey on echinococcosis in Darlag County of Qinghai Province]. Zhongguo Ji Sheng Chong Xue Yu Ji Sheng Chong Bing Za Zhi 27, 22–26.

Li, T., Chen, X., Zhen, R., Qiu, J., Qiu, D., Xiao, N., Ito, A., Wang, H., Giraudoux, P., Sako, Y., Nakao, M., Craig, P.S., 2010. Widespread co-endemicity of human cystic and alveolar echinococcosis on the eastern Tibetan Plateau, northwest Sichuan/southeast Qinghai, China. Acta Trop. 113, 248–256. doi:10.1016/j.actatropica.2009.11.006

Liu, H., He, D., Wu, X., Wang, H., Ma, X., Zhao, Y., 2008. [An epidemiological survey on hydatid disease in Yushu Prefecture of Qinghai Province]. Zhongguo Ji Sheng Chong Xue Yu Ji Sheng Chong Bing Za Zhi 26, 480–481, 484.

Luo, A., Wang, H., Li, J., Wu, H., Yang, F., Fang, P. 2014. Epidemic factors and control of hepatic echinococcosis in Qinghai province. J. Huazhong Univ. Sci. Technolog. Med. Sci. 34, 142–145. doi:10.1007/s11596-014-1246-8

Schantz, P.M., Wang, H., Qiu, J., Liu, F.J., Saito, E., Emshoff, A., Ito, A., Roberts, J.M., Delker, C., 2003. Echinococcosis on the Tibetan Plateau: prevalence and risk factors for cystic and alveolar echinococcosis in Tibetan populations in Qinghai Province, China. Parasitology 127 Suppl, S109–120.

Tiaoying, L., Jiamin, Q., Wen, Y., Craig, P.S., Xingwang, C., Ning, X., Ito, A., Giraudoux, P., Wulamu, M., Wen, Y., Schantz, P.M., 2005. Echinococcosis in Tibetan populations, western Sichuan Province, China. Emerg. Infect. Dis. 11, 1866–1873. doi:10.3201/eid1112.050079

Wang, Q., Huang, Y., Huang, L., Yu, W., He, W., Zhong, B., Li, W., Zeng, X., Vuitton, D.A., Giraudoux, P., Craig, P.S., Wu, W., 2014. Review of risk factors for human echinococcosis prevalence on the Qinghai-Tibet Plateau, China: a prospective for control options. Infect. Dis. Poverty 3, 3. doi:10.1186/2049-9957-3-3

Wang, Q., Qiu, J., Yang, W., Schantz, P.M., Raoul, F., Craig, P.S., Giraudoux, P., Vuitton, D.A., 2006. Socioeconomic and behavior risk factors of human alveolar echinococcosis in Tibetan communities in Sichuan, People’s Republic of China. Am. J. Trop. Med. Hyg. 74, 856–862.

Wang, Q., Huang, Y., Huang, L., Yu, W., He, W., Zhong, B., Li, W., Zeng, X., Vuitton, D.A., Giraudoux, P., Craig, P.S., Wu, W., 2014. Review of risk factors for human echinococcosis prevalence on the Qinghai-Tibet Plateau, China: a prospective for control options. Infect. Dis. Poverty 3, 3. doi:10.1186/2049-9957-3-3

Wang, Y.H., Rogan, M.T., Vuitton, D.A., Wen, H., Bartholomot, B., Macpherson, C.N., Zou, P.F., Ding, Z.X., Zhou, H.X., Zhang, X.F., Luo, J., Xiong, H.B., Fu, Y., McVie, A., Giraudoux, P., Yang, W.G., Craig, P.S., 2001. Cystic echinococcosis in semi-nomadic pastoral communities in north-west China. Trans. R. Soc. Trop. Med. Hyg. 95, 153–158.

Wang, Z., Wang, X., Liu, X., 2008. Echinococcosis in China, a review of the epidemiology of Echinococcus spp. EcoHealth 5, 115–126. doi:10.1007/s10393-008-0174-0

Xu, G.-R., Zhang, L.-J., Zeng, G., 2013. [Epidemic analysis of echinococcosis in Ganzi Tibetan Autonomous Prefecture of Sichuan Province from 2006 to 2011]. Zhongguo Ji Sheng Chong Xue Yu Ji Sheng Chong Bing Za Zhi 31, 224–228.

Yang, Y.R., Cheng, L., Yang, S.K., Pan, X., Sun, T., Li, X., Hu, S., Zhao, R., Craig, P.S., Vuitton, D.A., McManus, D.P., 2006a. A hospital-based retrospective survey of human cystic and alveolar echinococcosis in Ningxia Hui Autonomous Region, PR China. Acta Trop. 97, 284–291. doi:10.1016/j.actatropica.2005.12.001

Yang, Y.R., Craig, P.S., Ito, A., Vuitton, D.A., Giraudoux, P., Sun, T., Williams, G.M., Huang, Z., Li, Z., Wang, Y., Teng, J., Li, Y., Huang, L., Wen, H., Jones, M.K., McManus, D.P., 2007. A correlative study of ultrasound with serology in an area in China co-endemic for human alveolar and cystic echinococcosis. Trop. Med. Int. Health TM IH 12, 637–646. doi:10.1111/j.1365-3156.2007.01834.x

Yang, Y.R., Craig, P.S., Sun, T., Vuitton, D.A., Giraudoux, P., Jones, M.K., Williams, G.M., McManus, D.P., 2008. Echinococcosis in Ningxia Hui Autonomous Region, northwest China. Trans. R. Soc. Trop. Med. Hyg. 102, 319–328. doi:10.1016/j.trstmh.2008.01.007

Yang, Y.R., Sun, T., Li, Z., Zhang, J., Teng, J., Liu, X., Liu, R., Zhao, R., Jones, M.K., Wang, Y., Wen, H., Feng, X., Zhao, Q., Zhao, Y., Shi, D., Bartholomot, B., Vuitton, D.A., Pleydell, D., Giraudoux, P., Ito, A., Danson, M.F., Boufana, B., Craig, P.S., Williams, G.M., McManus, D.P., 2006b. Community surveys and risk factor analysis of human alveolar and cystic echinococcosis in Ningxia Hui Autonomous Region, China. Bull. World Health Organ. 84, 714–721.

Yu, S.-H., Wang, H., Wu, X.-H., Ma, X., Liu, P.-Y., Liu, Y.-F., Zhao, Y.-M., Morishima, Y., Kawanaka, M., 2008. Cystic and alveolar echinococcosis: an epidemiological survey in a Tibetan population in southeast Qinghai, China. Jpn. J. Infect. Dis. 61, 242–246.

3. Mongolia is highly endemic for *Echinococcus granulosus*. However there are no available data for the incidence of the disease. The reports listed below indicate it is a commonly encountered disease in Mongolia. As it is a post soviet state with a larger pastoral population it is likely to have a similar incidence to the former Soviet States in central Asia.

**References**

Garin-Bastuji, B., 1999. Report of a mission to Mongolia from 2 to 16 April 1999, on the Epidemiological surveillance and control of communicable diseases of public health importance, including zoonoses. World Health Organisation.

Gurbadam, A., Nyamkhuu, D., Nyamkhuu, G., Tsendjav, A., Sergelen, O., Narantuya, B., Batsukh, Z., Battsetseg, G., Oyun-Erdene, B., Uranchimeg, B., Otgonbaatar, D., Temuulen, D., Bayarmaa, E., Abmed, D., Tsogtsaikhan, S., Usukhbayar, A., Smirmaul, K., Gereltuya, J., Ito, A., 2010. Mongolian and Japanese Joint Conference on “Echinococcosis: diagnosis, treatment and prevention in Mongolia” June 4, 2009. Parasit. Vectors 3, 8. doi:10.1186/1756-3305-3-8
